# Lung function measurements in preclinical research: What has been done and where is it headed?

**DOI:** 10.3389/fphys.2023.1130096

**Published:** 2023-03-22

**Authors:** Kaveh Ahookhosh, Jeroen Vanoirbeek, Greetje Vande Velde

**Affiliations:** ^1^ Biomedical MRI, Department of Imaging and Pathology, KU Leuven, Leuven, Belgium; ^2^ Centre of Environment and Health, Department of Public Health and Primary Care, KU Leuven, Leuven, Belgium

**Keywords:** pulmonary function tests, non-invasive tests, invasive tests, pulmonary functional imaging, imaging-based techniques

## Abstract

Due to the close interaction of lung morphology and functions, repeatable measurements of pulmonary function during longitudinal studies on lung pathophysiology and treatment efficacy have been a great area of interest for lung researchers. Spirometry, as a simple and quick procedure that depends on the maximal inspiration of the patient, is the most common lung function test in clinics that measures lung volumes against time. Similarly, in the preclinical area, plethysmography techniques offer lung functional parameters related to lung volumes. In the past few decades, many innovative techniques have been introduced for *in vivo* lung function measurements, while each one of these techniques has their own advantages and disadvantages. Before each experiment, depending on the sensitivity of the required pulmonary functional parameters, it should be decided whether an invasive or non-invasive approach is desired. On one hand, invasive techniques offer sensitive and specific readouts related to lung mechanics in anesthetized and tracheotomized animals at endpoints. On the other hand, non-invasive techniques allow repeatable lung function measurements in conscious, free-breathing animals with readouts related to the lung volumes. The biggest disadvantage of these standard techniques for lung function measurements is considering the lung as a single unit and providing only global readouts. However, recent advances in lung imaging modalities such as x-ray computed tomography and magnetic resonance imaging opened new doors toward obtaining both anatomical and functional information from the same scan session, without the requirement for any extra pulmonary functional measurements, in more regional and non-invasive manners. Consequently, a new field of study called pulmonary functional imaging was born which focuses on introducing new techniques for regional quantification of lung function non-invasively using imaging-based techniques. This narrative review provides first an overview of both invasive and non-invasive conventional methods for lung function measurements, mostly focused on small animals for preclinical research, including discussions about their advantages and disadvantages. Then, we focus on those newly developed, non-invasive, imaging-based techniques that can provide either global or regional lung functional readouts at multiple time-points.

## 1 Introduction

Lungs play the most important role in the gas exchange process by transferring oxygen from the inhaled air to blood. Any chronic pulmonary abnormality eventually causes morphological destructions in the lung, which consequently reflect as pulmonary functional changes (Hoymann, 2007). Due to this close relationship between lung morphology and function, accurate measurement of lung function at multiple time-points is of great interest for diagnostic and prognostic purposes. Since the primary function of the lung is gas exchange, the pulmonary function can be characterized by ventilation, the distribution of the inhaled air into and out of the alveoli, and perfusion, the flow of blood to alveolar capillaries (Powers and Dhamoon, 2019).

Pulmonary function tests (PFTs) are not only valuable in the clinical context, but also in an experimental context which they are of utmost importance. Animal models are extremely crucial for gaining deeper insight into the cellular and molecular mechanisms involved in the pathogenesis of pulmonary diseases, simply because they allow experiments that are not authorized with humans (Nemery et al., 1987; Bates and Irvin, 2003). The principles controlling ventilation, airflow, lung volume, and gas exchange are almost the same among most of the mammals (Costa and Tepper, 1988; Hoymann, 2007; Hoymann, 2012). During the past decades, the growing interest in longitudinal lung functional studies on rodents led to an exploration for finding novel, more sensitive, non-invasive methods for repeated pulmonary function measurements (Glaab et al., 2007; Hoymann, 2007; Bates, 2017). This search resulted in introducing many invasive and non-invasive techniques for obtaining lung functional data during longitudinal animal studies in different lung research areas, such as pharmacological efficacy studies, safety pharmacological studies, and toxicological investigations (Bates and Irvin, 2003; Glaab et al., 2005; Hoymann, 2007; Hoymann, 2012; Bates, 2017). Each of these methods has their own advantages and disadvantages, which makes them suitable only for certain kinds of experiments (Glaab et al., 2007; de Andrade Castro and Russo, 2019). On one hand, invasive methods use anesthetized, paralyzed, tracheotomized animals, which are far from their natural conditions, however, they offer precise and specific readouts closely related to lung mechanics (De Vleeschauwer et al., 2011). On the other hand, non-invasive methods conveniently allow repeated pulmonary functional measurements in conscious animals with readouts related to the lung volumes, but with less sensitivity to pulmonary mechanics compared to the invasive methods (Hoymann, 2007). Therefore, based on the goal of the experiment, it should be decided whether functional readouts related to natural breathing patterns of conscious animals are required (non-invasive methods), or sensitive, accurate, and specific parameters related to lung mechanics (invasive methods).

These conventional methods of lung function measurements, invasive and non-invasive, only deliver global readouts. While, a wide range of lung diseases start locally by deteriorating lung parenchyma as well as small airways, and their functional effects are usually masked by lung compensatory mechanisms until significant sections of the lung structure are lost (Hsia, 2004; Burgel et al., 2013; Hsia, 2017; Stockley et al., 2017). Since small airways contribute minimally to airflow resistance, standard PFTs in clinics, such as spirometry and plethysmography which measure pulmonary functional parameters related to lung volumes cannot detect their loss at early stages. In the case of small airway diseases, such as COPD, fibrosis, emphysema, etc., these PFTs detect the lung function loss only after obstruction/destruction of 75% of the small airways (Cosio et al., 1978; Burgel et al., 2013). Therefore, the site of these small airways, approximately from the 8^th^ generation to terminal bronchioles and respiratory bronchioles, is called the “silent zone” (Burgel et al., 2013; Stockley et al., 2017). The biggest disadvantage of standard PFTs is that they consider the lung as a single unit, providing only global averages of functional parameters for the whole lung, which are not sensitive enough for early detection of most of the lung abnormalities (Ohno et al., 2022). This major limitation of the conventional PFTs prompted a search for techniques to acquire regional lung function data instead of global readouts. These techniques can be performed at experimental end-point, but are preferably non-invasive in the sense that the animals can fully recover from repeated functional measurements without any long-term injuries interfering with the experimental research question. Due to the recent advances of lung imaging modalities such as computed tomography (CT), magnetic resonance imaging (MRI), and nuclear medicine techniques, a new concept has emerged called “pulmonary functional imaging”, which utilizes imaging-based techniques to regionally measure lung functions (Gefter et al., 2021; Ohno et al., 2021). Pulmonary functional imaging with ability to provide regional lung functional data significantly improves our ability to detect and longitudinally evaluate many chronic pulmonary diseases at early stages. In the past few decades, several non-invasive techniques have been proposed for pulmonary functional imaging using CT, MRI, and nuclear medicine for clinical applications (Gefter et al., 2021; Ohno et al., 2021; Kooner et al., 2022; Ohno et al., 2022). Due to the importance of animal models in the understanding of pathogenesis of pulmonary diseases, these non-invasive, imaging-based techniques for regional lung function measurements are equally important for preclinical lung research.

In this narrative review, we describe and discuss both conventional and state-of-the-art experimental methods for lung function measurements focusing on small animals for preclinical and basic lung research. These methods fall roughly into two major categories, namely invasive, *i.e*. end-point measurements and non-invasive methods that can be applied repeatedly in the same animal, with or without a short anesthesia period. We further divide the non-invasive methods into imaging-based and non-imaging-based techniques. Then, we focus on those newly developed, non-invasive, imaging-based techniques that can provide either global or regional lung functional readouts at multiple time-points. We conclude with a discussion about future perspective of PFTs for longitudinal animal studies in biomedical research.

## 2 The role of pulmonary function tests in preclinical lung research

Murphy DG (2002) described the function of the respiratory system as a pumping apparatus, which includes nervous and muscular components, and a gas exchange unit (Murphy, 2002). While defects in the pumping apparatus can disrupt the breathing pattern, structural changes in airways, alveoli, and interstitial tissues including blood and lymph vessels that form the gas exchange unit lead to obstructive or restrictive diseases. Therefore, any change in pulmonary function detected by the standard PFTs stems from either disruption in pulmonary ventilation, or alteration in the mechanical properties of lungs (Murphy, 2002; Hoymann, 2012). For capturing these pulmonary function changes during progression of respiratory disorders, various invasive and non-invasive methods have been introduced throughout the past decades. These PFTs offer different lung functional parameters with different levels of sensitivities, with each one of these methods fitting to certain research questions. In the following subsections, we provide a detailed overview of both invasive and non-invasive PFTs for lung function measurements mostly in rodents, as well as discussions about their advantages and disadvantages that make them suitable for certain kinds of *in vivo* experiments.

### 2.1 Invasive methods for lung function measurements

Under invasive techniques for lung function measurements, we consider those methods that require the animals to be either orotracheally intubated (Likens and Mauderly, 1982; Brown et al., 1999; Glaab et al., 2004) or intubated *via* tracheostomy (Palecek et al., 1967), while breathing spontaneously or being mechanically ventilated during the procedure (Jackson and Watson, 1982; Schuessler and Bates, 1995). As this will lead to long-term injury, in practice, these methods are mostly considered as end-point measurements, carried out under terminal anesthesia compared to non-invasive methods, the value of invasive PFTs and pulmonary maneuvers lies in that they are far more sensitive for detecting those obstructive/restrictive lung disorders that change the mechanical properties of the gas exchange units.

#### 2.1.1 Dynamic compliance and lung resistance with plethysmography

Measurement of parameters such as dynamic compliance ( $C_{dyn}$ ) and lung resistance ( $R_{L}$ ) using invasive lung function measurements is a classical approach to determine pulmonary mechanics and airway responsiveness (Glaab et al., 2007). For the first time, in 1988, Martin et al. showed the viability of measuring these two parameters, $C_{dyn}$ and $R_{L}$ , in anesthetized, tracheotomized, and ventilated mice using body plethysmography (Martin et al., 1988). In this study, for evaluation of the bronchoconstrictor responses of normal C57BL/6 mice to bronchoconstrictor agonists, the authors connected the tracheotomized mice placed in a plethysmograph chamber to a pressure transducer and ventilator, where the device was set to provide 150 breathes/min with tidal volumes of 5–6 ml/kg. Lung volume changes of the mice were recorded by the plethysmograph using the pressure changes inside the chamber, which alongside the transducer signal and flow information were used to calculate the pulmonary compliance and resistance by the method of Amdur and Mead. (1958), that relates the tidal volume and the flow rate to intrapleural pressure at specific points during the respiratory cycle information to acquire the mechanical properties of the lungs. Followed by this pioneering technique, many more methods have been reported for measurement of $C_{dyn}$ and $R_{L}$ in anesthetized, tracheotomized mice using body plethysmography (Takeda et al., 1997; Taube et al., 2002; Irvin and Bates, 2003). In an attempt for repetitive measurements of $C_{dyn}$ and $R_{L}$ in mice, Brown et al. proposed a rapid, repeated intubation technique for anesthetized mice instead of tracheotomy (Brown et al., 1999). In this technique, the animal should be suspended at a 45° angle using a plexiglass support, while a light source illuminated the trachea below the vocal cord for better visualization. For a better view of the tracheal opening, a metal laryngoscope was used to keep the mouth open and hold the tongue out of the way to intubate the animal with a catheter attached to the hub of a needle. In this investigation, the authors have studied neither the maximum number of repeated intubations nor the timing between them which left doubts about the feasibility of the proposed method for repetitive measurements of $C_{dyn}$ and $R_{L}$ in anesthetized, instrumented mice (Brown et al., 1999).

In general, pulmonary compliance (C) can be defined as a parameter for measurement of lung expansion per each unit increase in the transpulmonary pressure, which can be divided into static and dynamic compliances (Marshall, 1957; Desai and Moustarah, 2020). While static compliance ( $C_{stat}$ ) represents pulmonary compliance when there is a fixed volume and no airflow, dynamic compliance describes the compliance during breathing and it monitors both elastic and airway resistance (Desai and Moustarah, 2020). Since certain respiratory disorders such as pulmonary fibrosis, emphysema, COPD, atelectasis, and newborn respiratory distress syndrome directly change the elastic properties of lung parenchyma, monitoring the compliance curve can be helpful to determine their progression (Lu and Rouby, 2000). In general, important factors such as elasticity of lung parenchyma, surface tension, surfactant, lung volume, smooth muscle contraction, and peripheral airway inhomogeneity can be considered as direct determinants of pulmonary compliance (Glaab et al., 2007; Desai and Moustarah, 2020). $R_{L}$ , which represents both airway and tissue resistance, is a dynamic force against the tracheobronchial tree and to some extend parenchyma deformation, which reflects both narrowing of the conducting airways and parenchymal viscosity (Glaab et al., 2007). Airway resistance ( $R_{aw}$ ), which can be described as the ratio between the pressure drop across the airway tree and the resulting airflow, highly depends on the geometry of the airway tree and the viscosity of the resident gas (Czovek, 2019). As the other contributor to lung resistance, tissue resistance ( $R_{ti}$ ), is a fundamental characteristic that is highly related to the elastic property of the tissue (Czovek, 2019). Asthma, COPD, cystic fibrosis, emphysema, and airway tumors are common pathological conditions that increase lung resistance (Özdilek, 2022).

Airway resistance and dynamic compliance are widely considered gold-standard parameters for diagnosis and quantification of bronchoconstriction and obstruction (Glaab et al., 2005; Hoymann, 2007; Ewart et al., 2010). The sensitivity and specificity of these parameters make them ideal choices for follow-up studies of testing safety of pharmacological compounds; however, despite many advantages, most of the approaches that measure pulmonary compliance and resistance require anesthetized and intubated/tracheotomized animals, which is far from the natural condition and mostly an endpoint for them (Bates and Irvin, 2003).

#### 2.1.2 Forced oscillation technique

The forced oscillation technique (FOT), for the first time introduced by DuBois et al. (1956), is a technique based on sinusoidal sound waves of a single frequency that pass through the lungs to provide information about pulmonary mechanics with parameters such as respiratory impedance (Zrs). Zrs is defined as the mechanical load of the respiratory system to ventilation (Navajas et al., 1991), and can be divided into resistance, which describes the resistance of conducting airways and tissue, and reactance (X), which reflects respiratory compliance and characterizes lung parenchyma (Glaab et al., 2007). For more insights into the basic concepts of FOT, we refer to (Pride, 1992; MacLeod and Birch, 2001; Oostveen et al., 2003; Tepper and Costa, 2015; Lundblad et al., 2021). To investigate the effects of drugs and diseases on pulmonary mechanics, FOT has been employed for measuring respiratory impedance both in rats (Jackson and Watson, 1982; Preuss et al., 1999) and mice (Schuessler and Bates, 1995; Vanoirbeek et al., 2010; Devos et al., 2017; Mori et al., 2017). Compared to the classical FOT approach for measuring pulmonary resistance and compliance, low-frequency forced oscillation technique (LFOT) provides even more details about pulmonary mechanics (Irvin and Bates, 2003; Peslin and Fredberg, 2011). In the case of LFOT, because a lower frequency sound wave travels further in the conducting airways and reaches smaller airways and lung parenchyma, it can provide more detailed information about lung mechanics (Brashier and Salvi, 2015). The biggest advantage of LFOT is the capability of showing differentiation between airway and tissue mechanics (Glaab et al., 2007).

The impulse oscillometry system (IOS), introduced by Michaelson et al., in 1975 using a computer-driven loudspeaker, is a FOT technique that utilizes multiple sound frequencies at the same time instead of a single frequency (Michaelson et al., 1975). The main advantage of employing multiple oscillation frequencies is that IOS calculates airway resistance in a way that allows differentiation between the behavior of large and small airways. Nowadays, almost all of the commercialized devices for lung function measurements such as FlexiVent [SCIREQ^©^ (Inc, 2022)] employ multiple oscillation frequencies for pulmonary functional and mechanical assessments. In the case of any airway obstruction, either in the central or peripheral airways, the total airway resistance increases above the normal value (Brashier and Salvi, 2015). Therefore, LFOT and IOS are accurate and powerful techniques to measure parameters such as resistance, reactance, and consequently respiratory impedance over a range of frequencies. However, similar to invasive plethysmography techniques, implementing these techniques still requires anesthesia, intubation, and even a higher level of expertise in handling.

Similar to preclinical lung research, FOT measurements also play an important role in clinical practice for early detection of the effects of smoking and COPD (Goldman, 2001; Oostveen et al., 2003; Ribeiro et al., 2018; Bhattarai et al., 2020). FOT as a non-invasive technique for measuring respiratory mechanics is already approved after comparative studies with classical spirometry readouts (Dellacà et al., 2004; Faria et al., 2010; Amaral et al., 2013; Su et al., 2018). The modern clinical devices for FOT measurements are able to cover a wide range of frequencies, lower than 5 Hz to assess peripheral airways and higher than 20 Hz to measure proximal airway resistance, which allows independent evaluation of proximal and peripheral airways (Shinke et al., 2013; Contoli et al., 2016; Berger, 2018). For low frequency measurements, i.e. less than 5 Hz, loudspeakers, a piston-type mechanical device (Kaczka et al., 1997), or pneumatic proportional solenoid valves (Kaczka and Lutchen, 2004) are used. Despite the advantages of FOT measurements, more clinical studies are required for correct interpretation of parameters such as elastance and reactance that highly depend on frequency (King et al., 2020).

#### 2.1.3 Forced pulmonary maneuvers

**FIGURE 1:**
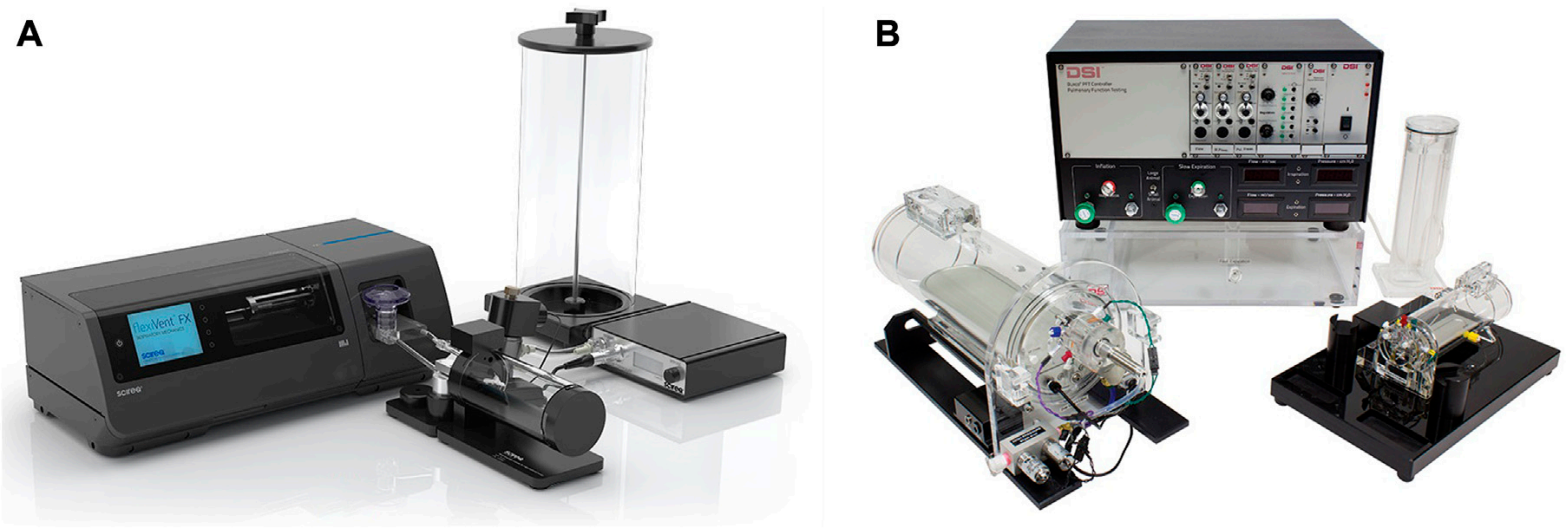
Commercially available experimental setups for *in vivo* lung function measurements in small animals **(A)** FlexiVent [reprinted from www.scireq.com (Inc, 2022)]; **(B)** Buxco [reprinted from www.datasci.com (Buxco, 2022)].

The two most commonly used commercially available devices for invasive lung function measurements in small animals are FlexiVent [SCIREQ^©^ (Inc, 2022)] and Buxco-forced pulmonary maneuvers [DSI^©^ (Buxco, 2022)] (Figure 1). These devices are widely considered the gold-standard for *in vivo* lung function measurements, since both of them are capable of performing forced oscillation technique, negative pressure-driven forced expiratory maneuvers (NPFE), and measuring standard pressure-volume (PV) curves. These techniques offer relevant parameters such as resistance, compliance, and elastance in anesthetized animals with high sensitivity and specificity. To delineate the existing potential of the invasive and non-invasive methods for lung function measurements, Vanoirbeek et al. (2010) employed FlexiVent and Buxco systems, as well as unrestrained plethysmography to assess two well-established models lung disease: a model of elastase-induced pulmonary emphysema, and a model of bleomycin-induced pulmonary fibrosis. The invasive techniques, unlike unrestrained plethysmography, using lung functional parameters such as functional residual capacity, total lung capacity, vital capacity, and compliance of the respiratory system could effectively distinguish the pulmonary emphysema from fibrosis. They concluded that both invasive systems for lung function measurements are sensitive enough for monitoring lung pathologies, however, FlexiVent has the advantage of an in-line nebulizer for testing hyperreactivity with methacholine. However, these commercialized devices for lung function measurements share the same disadvantages as the previous invasive techniques, including the requirement for terminal anesthesia, intubation/tracheostomy, high level of expertise in handling, and ventilatory maneuvers instead of spontaneous breathing, which does not always reflect the physiological situation (de Andrade Castro and Russo, 2019). Furthermore, one animal at a time can be handled during each lung function measurement which makes the technique time-consuming in *vivo* animal studies. Among these, the most important drawback is that these are endpoint lung function measurements due to tracheostomy, however, attempts have been made to address this issue by replacing tracheostomy with intubation for repeated invasive lung function measurements (Glaab et al., 2004; Glaab et al., 2005; De Vleeschauwer et al., 2011; Bonnardel et al., 2019). In a recent attempt, Bonnardel et al. used the FlexiVent system to prove the feasibility of repeated lung function measurements by intubation of healthy BALB/cJ mice and C57BL/6J mice to obtain parameters such as forced vital capacity (FVC), compliance of respiratory system (Crs), and forced expiratory volume in the first 0.1 s (FEV0.1) (Bonnardel et al., 2019). The authors reported an accurate evaluation of FVC, Crs, and FEV0.1 for intubated BALB/cJ mice, and FVC, FEV0.1, and inspiratory capacity (A) for intubated C57BL/6J mice. Despite the efforts for showing the feasibility of performing repetitive invasive lung function measurements in small animals, they are not suitable for reproducibly repeated measurements, resulting in that commercially available devices are still routinely used for endpoint measurements only. The lack of an alternative, truly non-invasive method for reproducible, repetitive detailed lung function measurements warrants further investigations for finding a reliable method with the least invasiveness for obtaining detailed lung functional data with direct readouts related to lung mechanics.

### 2.2 Non-invasive methods for lung function measurements in rodents

For *in vivo* longitudinal investigations of pulmonary function as well as screening large numbers of conscious small animals, non-invasive approaches are a prerequisite. In the following sections, we divide the non-invasive conventional methods and state-of-the-art techniques for lung function measurements into imaging-based and non-imaging-based methods and discussed them in detail.

#### 2.2.1 Non-imaging-based techniques

##### 2.2.1.1 Unrestrained whole-body plethysmography

Non-invasive plethysmography techniques offer lung function readouts of conscious animals longitudinally. Unrestrained whole-body plethysmography (UWBP), as an extreme of non-invasiveness, provides lung function data of several awake mice/rats at the same time and at several time points. Basically, the animals are placed each into a separate closed chamber to record breathing-induced oscillations of pressure inside the chamber by employing a barometric analysis technique. This technique provides parameters such as tidal volume and respiratory frequency (Glaab et al., 2007; Vanoirbeek et al., 2010; Bates, 2017). UWBP has been employed by many researchers for longitudinal measurement of lung functions in spontaneously breathing animals, especially by reporting a parameter called enhanced pause (Penh) (Hamelmann et al., 1997; Chong et al., 1998; Finotto et al., 2001; Donaldson et al., 2002). Penh is a dimensionless parameter used for the evaluation of changes in the shape of the airflow entering and leaving a whole-body plethysmograph (Bates, 2017). In 1997, for the first time using UWBP, a study on airway hyperresponsiveness (AHR) using aerosolized methacholine challenge in conscious, spontaneously breathing mice, revealed a good correlation between Penh and readouts such as lung resistance obtained from an invasive method (Hamelmann et al., 1997). Following this pioneering study, a few more investigations confirmed good correlations between Penh and gold-standard functional parameters obtained from invasive lung function measurements (Chong et al., 1998; Berry et al., 1999; Finotto et al., 2001; Donaldson et al., 2002; Kumar et al., 2004). However, further experiments have raised serious uncertainties and questions such as: what Penh as a dimensionless index really measures and to what extent it can be related to lung mechanics (Lundblad et al., 2002; Mitzner and Tankersley, 2003; Adler et al., 2004; Bates et al., 2004; Sly et al., 2005). Using unrestrained plethysmography investigating the relationship between Penh and lung resistance, it has been shown that UWBP can determine lung resistance only when tidal volume and functional lung capacity are measured independently. Also, humidity and temperature of the gas inside the chamber should be preconditioned to the animal’s body (Lundblad et al., 2002). Since fulfilling these conditions with conscious, unrestrained animals are not possible, the authors suggested that Penh should not be used for bronchial responsiveness assessments and sent a clear warning to the community that Penh cannot represent airway or pulmonary resistance. Followed by this enlightening study, more researchers (Adler et al., 2004; Bates et al., 2004) shared their serious concerns about replacing invasive mechanical indexes such as lung resistance with Penh, since this dimensionless parameter lacks the necessary physical principles. Despite intense criticism against the application of Penh to measure airway reactivity and AHR, for further exploration of Penh, Lomask discussed the mathematics of unrestrained plethysmography for two types of whole-body plethysmographs, pressure (PWBP) and flow (FWBP) plethysmographs (Lomask, 2006). The author confirmed that PWBP that utilizes a sealed chamber poorly correlates with airway resistance at room temperature. However, the Penh values obtained by FWBP that uses a chamber with a pneumotachograph correlate with resistance. Similarly, the relationships between Penh and thoracic airflow patterns have shown that Penh of plethysmography airflow is a sensitive indicator of an increase in specific airway resistance (Frazer et al., 2011). Nevertheless, UWBP as a convenient, quick, and non-invasive method can be employed for gross screening of overall ventilatory function in rodents. However, considering the cautionary warnings toward the misusage of Penh, especially in lung studies on airway responsiveness, ventilatory function obtained from UWBP should be corroborated with independent direct measurements of pulmonary mechanics (Vanoirbeek et al., 2004; Vanoirbeek et al., 2006; Hoymann, 2007; Tarkowski et al., 2007).

##### 2.2.1.2 Unrestrained video-assisted plethysmography

Since lung function data obtained from unrestrained plethysmography have no direct link to the mechanical properties of the lung, Bates et al. (2008) introduced unrestrained video-assisted plethysmography (UVAP) to non-invasively determine lung mechanical function in small animals. Reliable measurement of lung mechanical function requires a precise assessment of lung volume changes during the animal’s breathing, which is beyond the capability of UWBP (Lundblad et al., 2002; Adler et al., 2004; Bates et al., 2004). However UVAP, as an extension of UWBP, was an attempt to more precisely estimate lung volume using orthogonal video imaging (Bates et al., 2008). In UWBP, the measurements are based on the chamber pressure fluctuations due to the animal’s breathing, which results from the fact that the change in lung volume is not equal to the volume of inspired air from the chamber. Two physical processes can be introduced as the reason for this difference (Mitzner and Tankersley, 2003; Adler et al., 2004; Bates et al., 2008): (Hoymann, 2007) During inspiration, the respiratory musculature produces a necessary pressure gradient that drives the inspired air through the resistive airways, which also leads to thoracic gas compression; (Powers and Dhamoon, 2019); Due to the different temperature and humidity inside the thorax compared to the chamber, the inspired air expands inside the lungs. It has been shown that the pressure change due to the gas conditioning inside the lungs can be eliminated by heating and humidifying the air inside the plethysmography chamber to match the condition inside the lungs (Lundblad et al., 2002). Therefore, by preconditioning the air inside the chamber, the pressure fluctuations during the animal’s breathing can be related directly to the thoracic gas compression, which is also influenced by tidal volume (Bates et al., 2008). The constructed plethysmograph was a cuboidal chamber with two clear orthogonal sides for monitoring the animal, and a water jacket on the remaining sides for controlling the temperature inside the chamber. The humidity of the chamber was also controlled continuously by introducing a stream of air to the chamber after passing over a flask of hot water, except for those brief moments when lung function measurements were acquired, and the chamber was completely sealed. A pressure transducer was utilized for assessment of the pressure inside the chamber relative to the atmospheric pressure and two video cameras were fixed close to the plethysmography chamber to monitor the two orthogonal sides. Using this setup, the authors tried to simultaneously measure the pressure inside the chamber, as well as the changes in lung volume by assuming the animal’s body as an elliptical cross section in the acquired orthogonal silhouettes (Bates et al., 2008).

As the biggest advantage of this system, UVAP is able to directly and more precisely measure specific airway resistance in unrestrained and spontaneously breathing mice compared to UWBP. However, there are still downsides to this system (Reynolds and Frazer, 2011): (Hoymann, 2007) movements of the animals are problematic due to the slow sampling resolution (25 Hz, camera speed) of the cameras; (Powers and Dhamoon, 2019); controlling the conditions inside the chamber including temperature and humidity makes the system more complicated and even may induce stress to the animal. Due to the mentioned limitations, despite the solid theory related to lung mechanics behind the UVAP, this extension of unrestrained plethysmography was unable to replace the invasive methods for lung function measurements and never became a widely used method for lung mechanical function measurements in small animals.

##### 2.2.1.3 Acoustic whole-body plethysmography

Acoustic whole-body plethysmography (AWBP), similar to UVAP, attempts to measure tidal volume more accurately compared to UWBP. The acoustic plethysmograph proposed by Reynolds and Frazer included a main chamber, nozzle, speaker, microphone, and end stop assembly to change the volume, which represents a resonant cavity that operates at a frequency that depended on the volume of the cavity and also the dimensions of the nozzle (Reynolds and Frazer, 2006). During the breathing of the animal in the plethysmography chamber, the volume around the animal changes due to the thorax movements which influences the amplitude of the acoustic pressure inside the chamber. In this system, the acoustic pressure of the chamber is almost independent of the animal’s lung volume, due to the fact that the acoustic input impedance of the system is very large because of the large change in area from the chamber to the nasal opening (Reynolds and Frazer, 2006). Since the sound pressure level (SPL) of the plethysmograph has a direct relationship with the signal-to-noise ratio of volume measurements, the sensitivity of the AWBP can be increased with higher values of SPLs, which is tolerable for mice (Fay, 1988). The acquired acoustic pressure signal inside the chamber was related to tidal volume using a signal processing technique. Similar to UVAP, VWBP can directly measure specific airway resistance in unrestrained, spontaneously breathing animals. However, this system is susceptible to ambient noise frequencies near the excitation frequency (Reynolds and Frazer, 2011), making it impractical to use for precise assessment of lung volume changes during the animal’s breathing in a laboratory setting without acoustic insulation.

##### 2.2.1.4 Head-out body plethysmography

**FIGURE 2:**
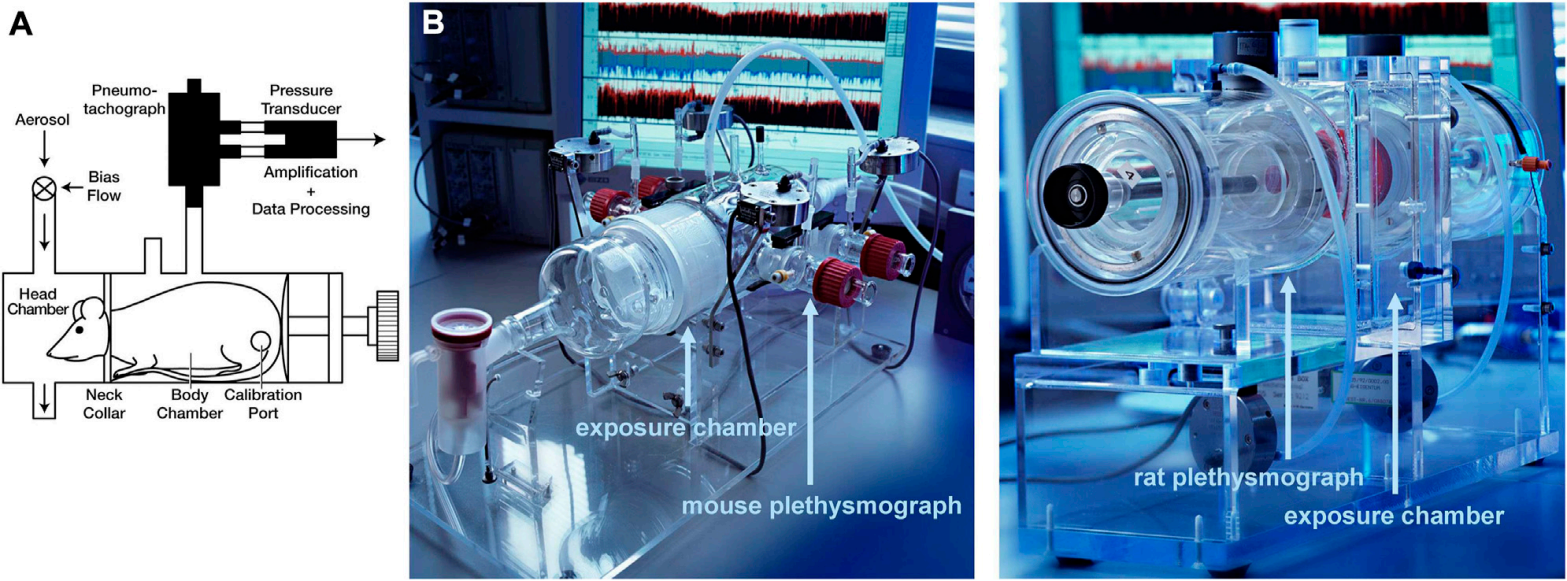
Head-out plethysmography **(A)** Schematic and **(B)** Photos of head-out plethysmography systems for mice and rats [reprinted from Hoymann. (2012)].

In head-out body plethysmography (Glaab et al., 2007), the head and body of the animal are separated by a seal in the plethysmograph, wherein the animal’s head is exposed to a continuous airflow in the head chamber, and the rest of the body is placed in the body chamber which is attached to a pressure transducer by a pneumotachograph tube (Figure 2). In the body chamber, the thoracic movements of the animal drive the flow to the pneumotachograph tube which finally reaches the differential pressure transducer, where the respiratory flow is measured and parameters such as respiratory rate and tidal volume are obtained (Hoymann, 2007). Commonly for employing this approach, the animals should be trained a few days before the lung function measurements to get used to the head-out plethysmograph (Hoymann, 2012). The introduction of head-out body plethysmography dates back to 1994, when Vijayaraghavan et al. non-invasively measured mid-expiratory flow ( ${EF}_{50}$ ) for the assessment of airway responsiveness in conscious mice (Vijayaraghavan et al., 1994). ${EF}_{50}$ is the midpoint of expiratory tidal volume, which can perfectly describe the main changes in tidal volume due to an airflow limitation caused by bronchoconstriction, edema, or accumulation of mucus (Glaab et al., 2007; Hoymann, 2012). Since then, many other research groups employed head-out plethysmography for examination of drug effects and proposed ${EF}_{50}$ as a meaningful, non-invasive parameter for determination of bronchoconstriction in mice and rats (Neuhaus-Steinmetz et al., 2000; Glaab et al., 2001; Glaab et al., 2002; Baelder et al., 2005; Glaab et al., 2005). In addition, validation studies by employing invasive and non-invasive PFTs showed good correlations between ${EF}_{50}$ and gold-standard functional parameters (Glaab et al., 2001; Glaab et al., 2002; Glaab et al., 2005). In 2005, Glaab et al. utilized head-out body plethysmography to non-invasively measure ${EF}_{50}$ in conscious mice which were exposed to inhalable *Aspergillus fumigatus* antigens, paralleled by invasive measurement of pulmonary conductance and dynamic compliance in anesthetized, orotracheally intubated mice (Glaab et al., 2005). The decrease in ${EF}_{50}$ and pulmonary conductance and dynamic compliance correlated well and despite the higher sensitivity of gold-standard parameters, ${EF}_{50}$ was sensitive enough to detect airway responsiveness in intact spontaneously breathing mice.

In conclusion, head-out body plethysmography is a non-invasive, simple, and repeatable method for lung function measurements that allows handling several conscious animals at the same time by attaching several chambers to a central system. Head-out body plethysmography offers valuable outputs such as ${EF}_{50}$ with physical meaning [ml/s] that directly relates to gold-standard pulmonary functional parameters such as airway resistance (Glaab et al., 2007). Despite its advantages, there is a risk of inducing the influence of stress to the results due to retainment of the animal during the measurements. However, this issue can be mitigated to some extent by training the animals beforehand and starting lung function measurements only after the animals settled down to a stable level (Glaab et al., 2007).

##### 2.2.1.5 Double-chamber plethysmography

Double-chamber plethysmograph, as the name explains, consists of two rigid chambers that separate the animal’s body from the neck to isolate the animal’s head and nose as hermetically as possible in the front chamber, from the rest of the body in the rear chamber (Figure 2). In the front chamber, where the restrained animal is consciously and spontaneously breathing, the produced flow from the nostrils is measured, while in the rear chamber the produced airflow by volume change due to the thorax movements is measured. Either using pressure transducers or pneumotachographs, waveform signals as a function of time from each chamber are recorded, which can finally produce respiratory parameters such as tidal volume and frequency (Reynolds and Frazer, 2011; Mailhot-Larouche et al., 2018). In addition to respiratory parameters, acquiring parameters such as ${EF}_{50}$ and specific airway resistance (sRaw), which are sensitive to airflow obstruction, is also possible with double-chamber plethysmography (Pennock et al., 1979; Neuhaus-Steinmetz et al., 2000; Glaab et al., 2001; DeLorme and Moss, 2002; Glaab et al., 2002; Flandre et al., 2003). While measurement of airway resistance requires both waveform signals obtained from head- and body-chambers, for calculating ${EF}_{50}$ , just like head-out body plethysmography, only the signal from body-chamber is needed. Therefore, depending on the application, double-chamber plethysmography can be employed with or without the head-chamber (Mailhot-Larouche et al., 2018). Regardless of the advantages, double-chamber plethysmography shares the same disadvantages as head-out plethysmography, which is the requirement of restraining the animal and facing the risk of inducing the effects of stress to the obtained results. Furthermore, the reproducibility of obtained parameters such as sRaw from double-chamber plethysmography for airway responsiveness has been challenged (Duguet et al., 2000; DeLorme and Moss, 2002). However, still many researchers suggest double-chamber plethysmography as a non-invasive, easy, rapid, and reproducible technique for longitudinal assessment of respiratory function in conscious animals after challenges with aerosolized substances (Lofgren et al., 2006; Mailhot-Larouche et al., 2018).

#### 2.2.2 Imaging-based techniques

All the lung function measurement techniques outlined so far more or less provide lung function readouts on diagnosis of lung diseases and severity in animal studies of lung diseases. The Flexivent and Buxco systems, applied as end-point measurements, provide the most detailed set of lung functional and mechanical readouts including parameters related to lung volumes, quasi static pressure-volume curves, as well as the capability to distinguish lung tissue properties from airway characteristics using the low-frequency forced oscillation technique (Shalaby et al., 2010; Vanoirbeek et al., 2010; De Vleeschauwer et al., 2011; Bates, 2017; de Andrade Castro and Russo, 2019). As such, they can differentiate between obstructive and restrictive lung diseases and can be considered as the gold standard tools for lung function assessments. Nevertheless, as these lung function measurements provide global readouts of lung and airway performance, they may underestimate the extent of lung pathology in cases where unaffected lung regions compensate for affected regions, such as in chronic respiratory diseases (CRDs), lung transplantation, and pneumonectomy (Wu et al., 2000; Dane et al., 2013; Ravikumar et al., 2013; Vos et al., 2014; Dekoster et al., 2020; Li et al., 2020). Therefore, the early diagnosis and longitudinal assessments of lung performance in different cases is currently stalled due to the inability to capture the complete spatial distribution of lung function. We would ideally need a tool that can provide regional readouts on lung function.

Imaging modalities such as CT and MRI as efficient visual tools can be employed not only to monitor lung structural changes, but also to obtain lung functional data. While non-imaging-based methods for lung function measurements only provide global readouts for lung function assessment, some of the imaging-based methods can longitudinally provide detailed regional data of lung performance, which may allow researchers to detect pulmonary diseases in early stages and test the therapeutics more effectively. On one hand, micro-CT with high spatial and temporal resolution offers a great potential to obtain detailed regional information about lung structure and function in alive animals, which can enable us to quantify the severity of pulmonary diseases at earliest

Stages in a non-invasive manner. While, the ability of micro-CT in longitudinal assessments of lung structural changes is already well-established in *vivo* lung disease studies (De Langhe et al., 2012; Poelmans et al., 2016; Dekoster et al., 2020), its great potential in providing regional pulmonary functional data still needs to be revealed. On the other hand, MRI without ionizing radiation and with employing hyperpolaraized gases can provide regional ventilation and perfusion maps. This section investigates the abilities of current lung imaging modalities for the regional/global assessment of lung function and biomechanics non-invasively in the presence of lung pathologies.

##### 2.2.2.1 X-ray computed tomography

**FIGURE 3:**
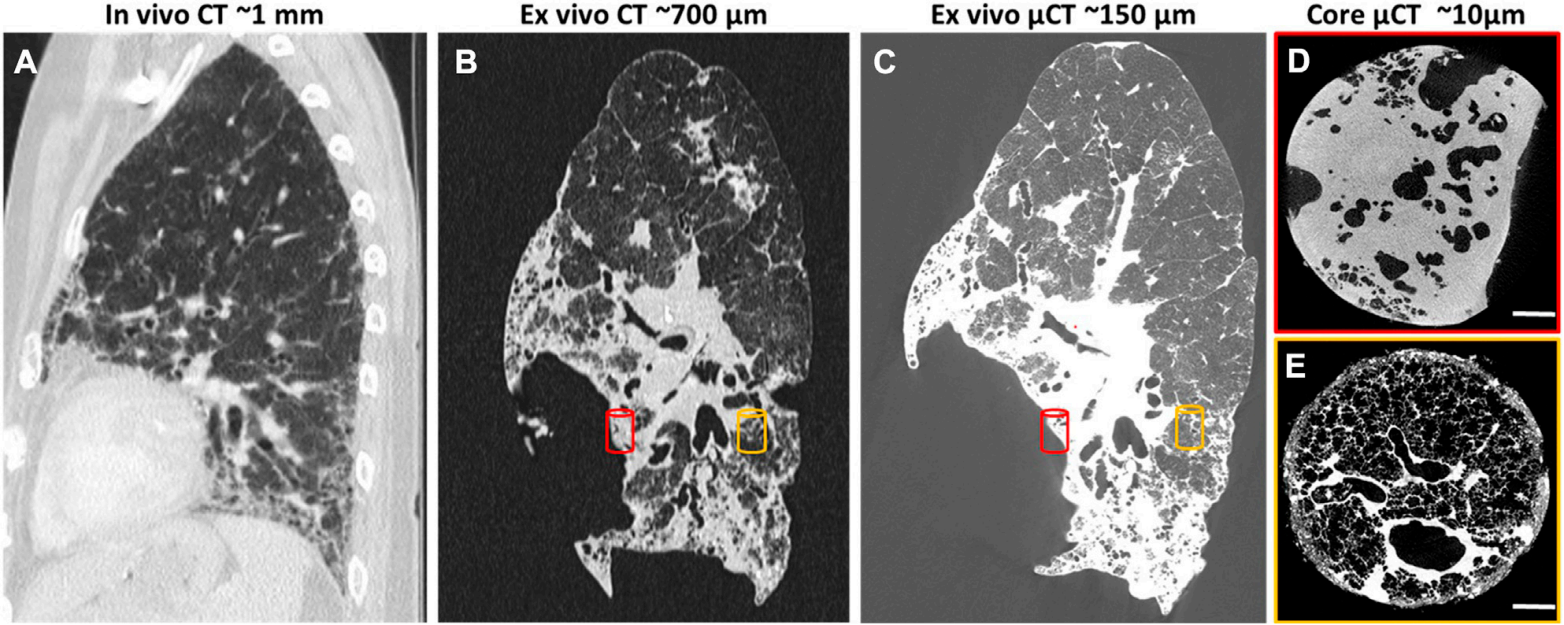
HRCT scans of human lung with usual interstitial pneumonia (UIP)[reprinted with permission from Tielemans et al. (2020)] **(A)** *In vivo* HRCT scan of the lung with resolution of 1 mm 6 months before lung transplantation; **(B)** *Ex vivo* CT scan of the lung after transplantation with resolution of 700 μm; **(C)** *Ex vivo* µCT scan of the same lung with higher resolution, 150 μm; **(D, E)** Core *ex vivo* µCT scan with resolution of 10 μm, showing severe fibrosis (red cylinder) and more healthy area (orange cylinder).

In clinics, X-ray CT as the gold-standard modality for lung medical imaging, plays an important role in the diagnostic and therapeutic workup of many lung diseases, due to its extensive availability, speed, high-resolution, and high signal-to-noise ratio for lung tissue (Simon, 2000; Tielemans et al., 2020). High-resolution computed tomography (HRCT) is the most updated optimized technique to acquire the most detailed lung images with a multidetector CT scanner (Verschakelen, 2010) (Figure 3). In addition to its traditional role in the diagnostic of diffuse parenchymal and interstitial lung diseases (DPILDs), CT techniques have been developed and utilized as non-invasive methods to measure regional ventilation in the lung for many years (Gur et al., 1979; Gur et al., 1981; Herbert et al., 1982; Snyder et al., 1984; Simon et al., 1998). Therefore, the significant potential of CT for developing new techniques has provided a magnificent opportunity to obtain not only detailed anatomical information on lung structure and pulmonary pathological patterns, but also quantitative functional characterization of the lung during the progression of pulmonary diseases (Young et al., 2019).

In preclinical research, µCT has proven to be a very powerful tool for longitudinal assessments of lung structural changes in chronic lung disease models, however, its routine implementation lags behind. Although, there are great opportunities to extract imaging-derived biomarkers not only on lung disease burdens, but also on lung function based on its ability to provide four dimensional (4D) data from different phases of the breathing cycle. Dynamic imaging of the lung using µCT to obtain 4D datasets is possible either with prospective synchronization (signal-based gating), triggering the image acquisition at certain phases of the respiratory cycle during scanning, or retrospective synchronization (image-based gating), sorting the acquired images according to their respiratory phases as a post-processing step (Liu et al., 2017). 4D-µCT of the lung using synchronization thereby not only reduces motion artifacts induced by cardiac and respiratory cycles, but also provides opportunities to extract end-inspiratory and end-expiratory images of the lungs, that can then be used to calculate (regional) tidal expansion (Ding et al., 2010; Vinogradskiy et al., 2012; Brennan et al., 2015). Using density measurements, several µCT-derived biomarkers such as mean lung density, total lung volume, areated and non-areated lung volumes have been introduced to longitudinally investigate the onset and progression of pulmonary diseases such as lung fibrosis, invasive pulmonary aspergillosis, and pulmonary cryptococcosis (De Langhe et al., 2012; Vande Velde et al., 2016; Dekoster et al., 2020). Furthermore, the distribution of air volume inside the lung can be obtained at several positive end-expiratory pressures (PEEPs) to plot a pressure-volume (P-V) curve, which can describe the static mechanical P-V relationship of the respiratory system with the assumption that the alveolar pressures are equilibrated (Marcucci et al., 2001). Calculating the distribution of regional air content and lung volume using density-based techniques has been employed by several researchers in a variety of applications to study topics such as post-pneumonectomy lung growth, special species adaptations, and respiratory distress syndrome (Hoffman and Ritman, 1985; Gattinoni et al., 1993; Olson and Hoffman, 1994; Gattinoni et al., 1995). The following sections investigate the x-ray-based techniques developed for lung function measurements in the recent years for preclinical lung research in more detail.

##### 2.2.2.2 Xenon-CT regional ventilation imaging

**FIGURE 4:**
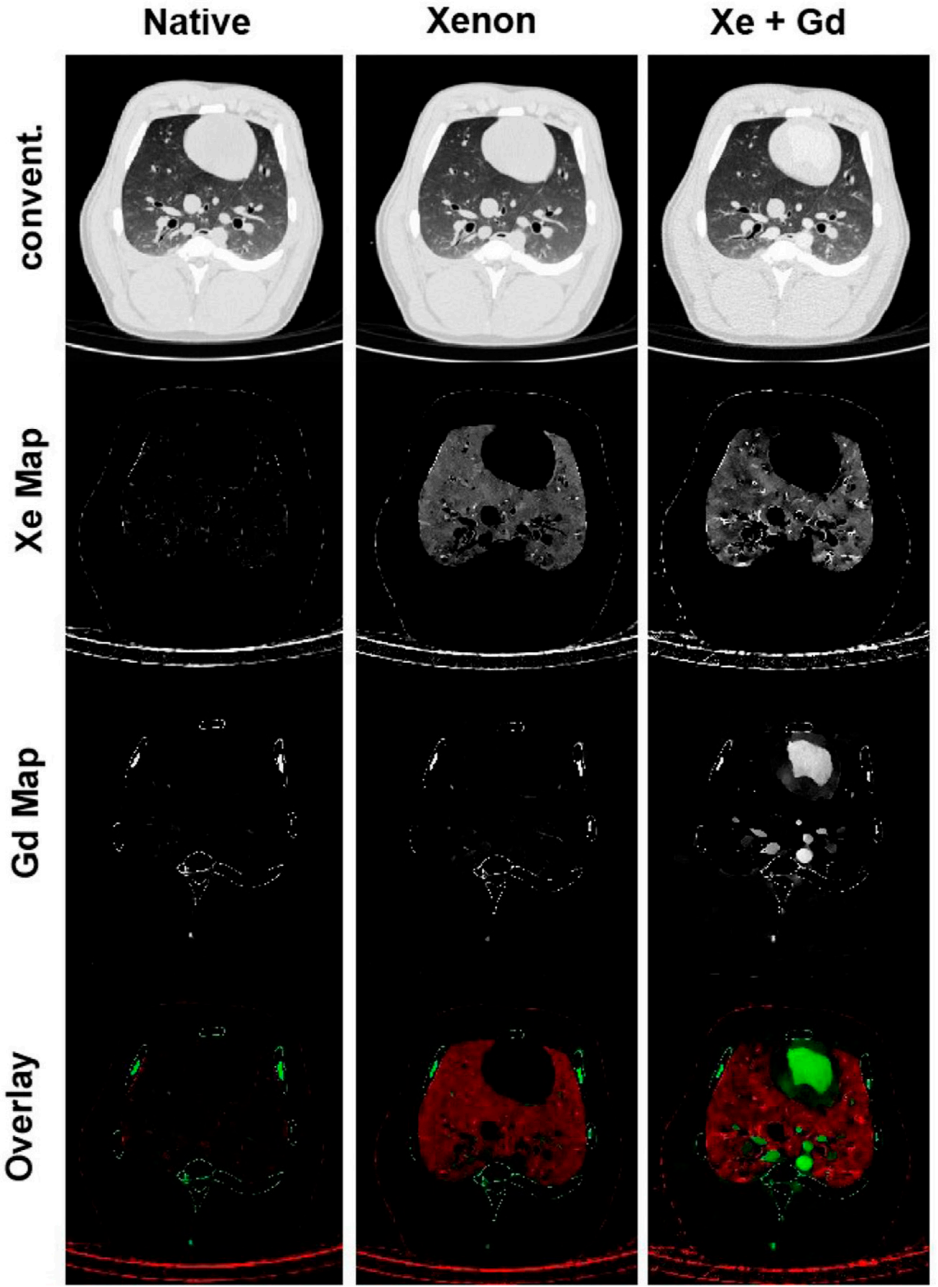
Xenon ventilation and gadolinium perfusion maps of a landrace pig acquired using dual-energy CT [reprinted from Sauter et al. (2019)]. Last row shows the overlay of the xenon and gadolinium density maps as a combined ventilation/perfusion maps.

Application of Xenon (Xe) gas as a contrast agent for CT-based regional ventilation measurements as a non-invasive procedure for evaluation of pulmonary function dates back to 1979, when Gur et al. used ventilation rate constants to discuss the pulmonary function of normal and impaired lungs (Gur et al., 1979). Since the density of Xe is higher than air, in the presence of this gas in the airways, the density of those areas in the CT images linearly increases with the Xe concentration; therefore, by serial scanning of the same ROI in the lung during wash-in and wash-out of Xe, regional ventilation can be mapped by density measurement techniques (Simon et al., 1998; Simon, 2005). Xe-CT ventilation imaging has been employed in many lung studies to non-invasively measure regional distributions of ventilation, perfusion, and ventilation/perfusion (V/Q) ratio (Figure 4) (Hoffman et al., 1995; Simon et al., 1998; Tajik et al., 1998; Marcucci et al., 2001; Jones et al., 2003; Sauter et al., 2019). However, despite many advantages such as providing high-resolution, regional ventilation maps non-invasively, Xe-CT method includes also several limitations. As an anesthetic gas, the concentartion of Xe cannot exceed 30%–35% for ventilation measurements in humans due to the side effects, which consequently limits the maximum CT density enhancement that can be acquired (Simon, 2000). Furthermore, since Xe is soluble in blood, the maximum alveolar concentration of this gas reduces during washing; while, most of the employed models for Xe-CT ventilation measurements consider no uptake and recirculation of Xe. In addition, due to the higher density and viscosity of Xe compared to air, the regional distribution of ventilation can be different from normal respiratory gases, especially at higher inspiratory flow rates (Simon, 2005).

##### 2.2.2.3 X-ray body plethysmography

X-ray body plethysmography was another attempt to cover the limitations of UWBP for accurate measurements of tidal and end-expiratory volumes using single projection x-ray imaging, with the purpose of assessing airway resistance in conscious, spontaneously breathing mice (Lai-Fook et al., 2008). The proposed plethysmograph by Lai-Fook et al. included a transparent plastic tube with a cone-shaped end which was connected to a thin-walled copper cylinder. The x-ray source and sensor were located in the plastic tube to acquire single projection images of the animal’s thorax using a single x-ray pulse of 10 ms exposure time to minimize image blur due to respiratory and cardiac cycles. The plethysmograph also included a heat lamp in the transparent plastic tube and a pressure transducer, thermistor, and humidity gauge in the thin-wall copper cylinder for controlling the air condition inside the chamber and measuring the pressure. Since evaluation airway resistance requires both tidal and end-expiratory volumes, single projection x-ray images and pressure oscillations inside the chamber were used to estimate these lung volumes in spontaneously breathing mice. Pressure oscillations inside the plethysmography chamber were assumed as sinusoidal variations. The biggest advantage of x-ray body plethysmography was the capability of measuring lung volumes in a way that allows separate estimations of airway resistance and compliance; however, the high cost of the x-ray system, slow collection of x-ray images throughout a breathing cycle, and the manual segmentation of the images, halted the way of seeing this system as an efficient screening tool (Bates et al., 2008; Reynolds and Frazer, 2011).

##### 2.2.2.4 X-ray lung function

**FIGURE 5:**
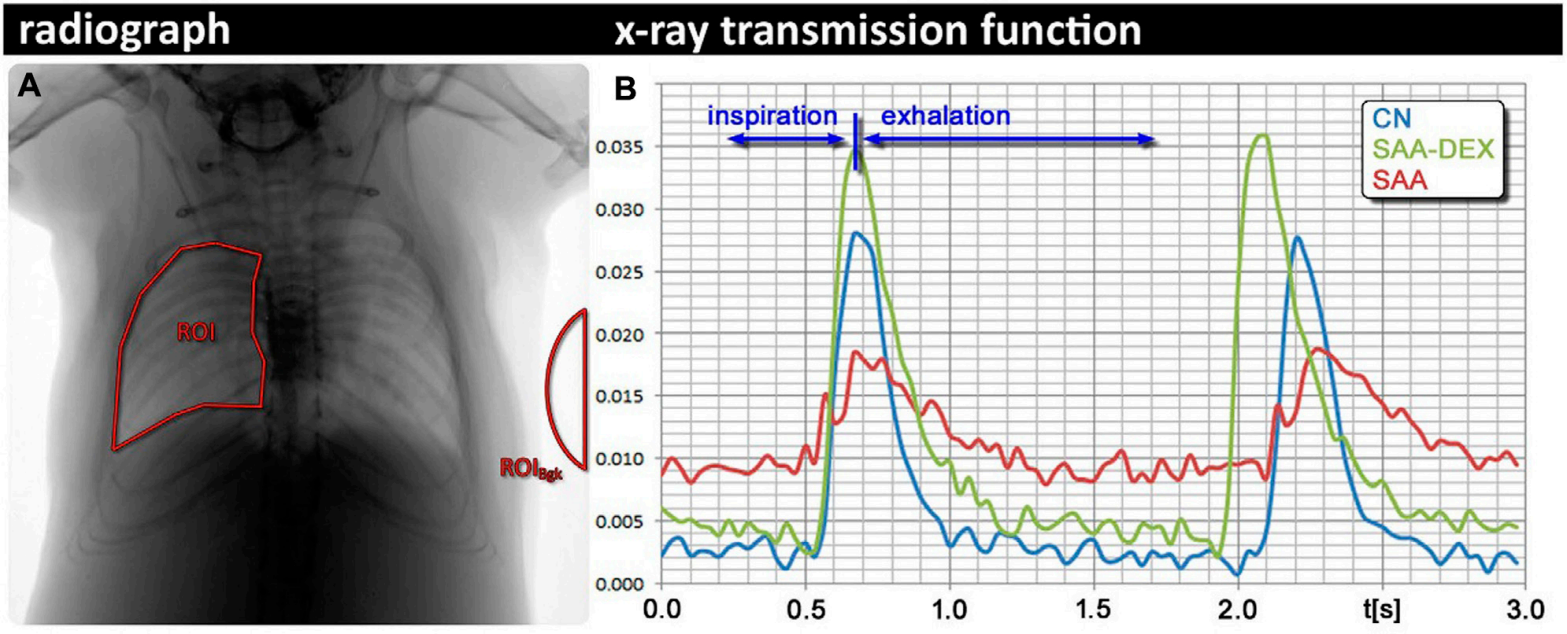
X-ray lung function (XLF) technique **(A)** Region of the interest of the lung in an exemplified radiograph. **(B)** Comparison between breathing cycles of a healthy control mouse (CN), a mouse with severe accute airway inflammation (SAA) 2 days after the last challenge, and a mouse from the same model, treated with dexamethasone before each challenge [reprinted from Dullin et al. (2016)].

In further exploration for finding a non-invasive method for lung function measurements and address drawbacks of plethysmography techniques, Dullin et al. (2016) introduced an imaging-based technique called x-ray lung function (XLF) method. XLF is a non-invasive technique for lung function measurements that employs low-dose planar cinematic x-ray imaging to monitor the animal’s breathing during the measurements. In this *in vivo* approach for lung function measurements, a video of 2D radiographs of the chest movements during spontaneously breathing of the unrestrained, anesthetized animal was captured to measure the average x-ray transmission of the lungs in each frame of the recorded video. The intensity fluctuations of the animal’s chest movements normalized by the average background signal in the acquired 2D movie were described using an x-ray transmission function (XTF) over time. After filtering, the breathing cycles of the XTF for each animal were parameterized using a third-order polynomial and the average of each parameter ( $b_{1}$ - $b_{5}$ ) over all of the breathing cycles were used to characterize the lung function by the ratio between inhalation and exhalation times as well as the maximum air content in the lung. Applying the XLF technique to an ovalbumin-induced experimental allergic airway disease mouse model mimicking severe acute asthma (SAA), Dullin et al. showed a significantly higher sensitivity for XLF in detecting the elasticity reduction of the lungs in comparison to UWBP (Dullin et al., 2016). XLF using the XTF parameters ( $b_{1}$ - $b_{5}$ ), showed shorter relative inspiration periods and reduced air flows in the lungs, which both pointed to a reduction in elasticity related to the inflammation in SAA. UWBP using parameters such as MaxSlope and MinSlope showed the same trend in air flow reduction for SAA mice compared to the controls. Furthermore, the authors also assessed the efficacy of dexamethasone as the common treatment of SAA (Figure 5), and correlated the results with UWBP, and further with post-mortem histology, broncho-alveolar lavage (BAL), and synchrotron phase contrast CT. Similarly, in comparison to UWBP, XLF with higher accuracy showed the improvement of lung function parameters in the treated mice with dexamethasone.

For further evaluation on reliability and sensitivity of XLF technique, the same research group used XLF and propagation synchrotron phase-contrast computed tomography (pSRμCT) for quantification of lung remodeling in an allergic airway inflammation (AAI) mouse model (Markus et al., 2017). In the lung function measurements using XLF, the breathing frequency during acquisition was adjusted to one breathing cycle in 1,400 msec using the level of anesthesia and overall for each mouse 21 breathing cycles were recorded. Using the same parameterization technique explained above, XTF parameters ( $b_{1}$ - $b_{5}$ ) were used to characterize the lung function of each animal. After each lung function measurement, the mice were euthanized for *in situ* lung imaging by synchrotron pSRμCT, to assess whether the XLF findings in the recovered mice are associated with subtle structural changes. Based on the pSRμCT results, the authors showed the persistence of airway remodeling after the resolution of the inflammatory response. In addition, they found a high degree of correlation between the pSRμCT volume ratio and XLF results, which showed a significant air trapping in AAI mice in comparison to controls, most probably due to the reduction of elasticity in the lungs induced by allergic exposure. The authors concluded that the persistent loss of lung elasticity in AAI mice even after a few months’ recovery can be related to the loss of elastic fibers. The results of this study once again confirmed the reliability and sensitivity of XLF as a non-invasive, *in vivo* technique for longitudinal lung function measurements in AAI mouse models.

**FIGURE 6:**
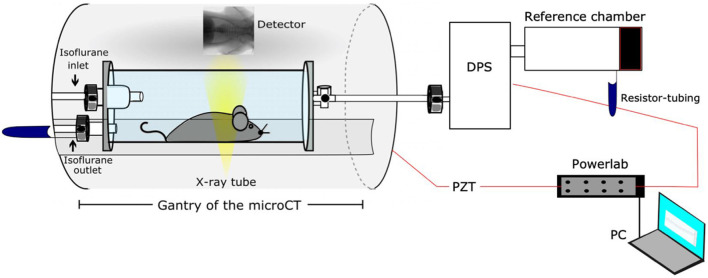
Schematic of the developed setup by Khan et al. for correlative XLF, WBP, and µCT measurements [reprinted from Khan et al. (Khan et al., 2021)].

Since none of the functional parameters ( $b_{1}$ - $b_{5}$ ) of XLF described pulmonary air volume, Dullin’s research group developed a unique experimental setup to simultaneously perform either XLF or µCT with WBP (Khan et al., 2021). They replaced the animal’s bed inside the gantry of the µCT scanner with a custom-made plethysmography chamber, which included a differential pressure sensor and isoflurane inlet-outlet for anesthesia (Figure 6). The original approach in XLF for calibration of x-ray transmission over time included a background selection for normalization, which required a large field of view (FOV) that led to low image resolution (Dullin et al., 2016). In this study, the XTF function was modified by applying a new approach for background correction, an adaptive moving filter, which provided the possibility of using a smaller FOV during the XLF measurements and consequently a better image resolution at the lung region (Khan et al., 2021). The relative x-ray transmission function (rXTF) was used for analysis of the breathing cycles and quantitative functional parameters such as end-inspiration lung volume (EIV), the relative x-ray transmission at end-expiration, as well as the decay rate of the expiration phase. The results showed a strong correlation between the acquired lung volume by rXTF function in XLF technique and those extracted from the µCT data; however, the XLF data were obtained by only 7% of the x-ray dose and 13% of the acquisition time used in µCT, which shows the capability of XLF as a reliable technique for *in vivo,* non-invasive lung volume measurements in longitudinal studies on lung diseases.

**FIGURE 7:**
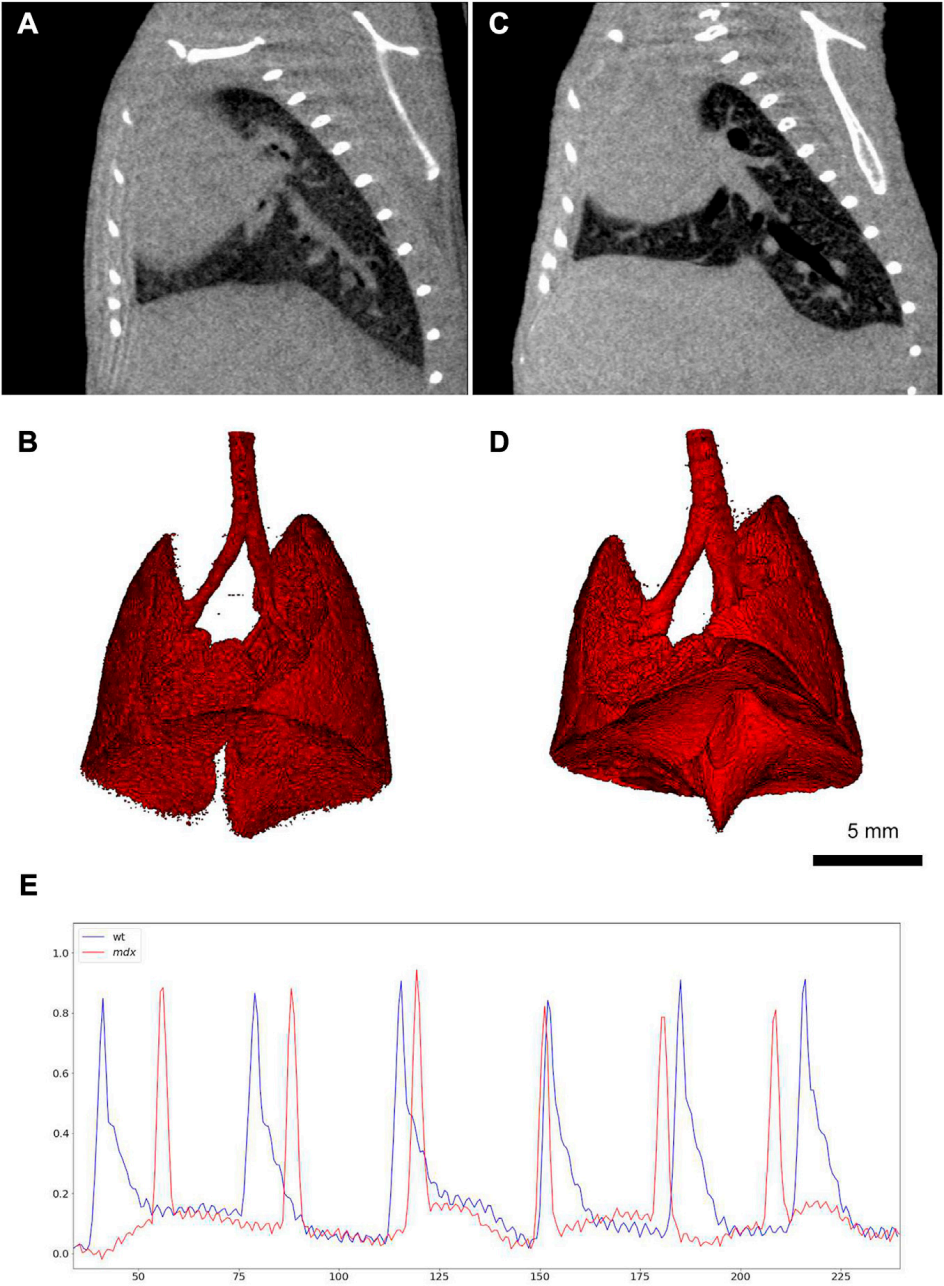
Retrospective gating-based x-ray lung function measurement (rgXLF) **(A, C)** µCT images of a healthy control mouse and a mdx mouse respectively, showing the difference in the shape of the diaphragms; **(B, D)** 3D renderings of the healthy and mdx mice; **(E)** Comparison of the breathing patterns of the healthy and mdx mice (healthy = blue and mdx = blue), showing a more rapid decay in the expiration phase of the mdx mouse [reprinted from Dullin et al. (2022)].

Since 2D radiographs of the chest movements are used by XLF protocol, the acquired imaging data could only be used for monitoring the animal’s breathing during the measurements and no anatomical information could be obtained. To address this issue, Dullin et al. proposed a new technique based on XLF to quantify lung function in the raw data of retrospectively gated lung µCT scans, which is called retrospective gating-based x-ray lung function measurement (rgXLF) (Dullin et al., 2022). For assessment of the newly developed technique, they applied the rgXLF protocol on mdx mice, the most commonly used mouse model for studying Duchenne muscular dystrophy (DMD). The authors employed the same strategy for parameterization of the breathing pattern that Khan et al. (Khan et al., 2021) used in their study. The comparison of functional parameters between XLF and rgXLF revealed a strong correlation, with almost the same k-values of the expiration phase and similar heart rates. In a comparison between control and mdx mice, both cross sections and 3D lung reconstruction showed the differences in the shape diaphragm between the mice (Figure 7). Therefore, rgXLF with a low x-ray dose, short acquisition time, and minimum voxel size of 40 µm showed the ability for longitudinal lung function measurements and also providing anatomical information without the requirement for additional scanning.

##### 2.2.2.5 Phase contrast X-ray lung function

The continuous search for finding new imaging-based techniques to obtain regional lung function and biomechanics data led to employing synchrotron radiation sources to produce highly coherent, high flux x-rays that are required for phase contrast x-ray imaging (PCXI) [see (Bayat et al., 2001), (Bayat et al., 2009), (Porra et al., 2004), (Monfraix et al., 2004), (Bayat et al., 2022a), (Cercos-Pita et al., 2022), (Bayat et al., 2022b)]. PCXI is a high-resolution imaging technique, capable of differentiating between soft tissues by enhancing the contrast of biological interfaces and also providing dynamic motions of lung tissue. Recently, Bayat et al. throughly reviewed the present methods for synchrotron radiation-based imaging that have been used for regional lung function measurements [see (Bayat et al., 2020)]. The synchrotron radiation-based imaging methods included free propagation-based phase-contrast lung imaging (PBI), speckle-based lung imaging, 4D lung imaging, and K-edge subtraction (KES) imaging as well as their applications in preclinical animal models.

**FIGURE 8:**
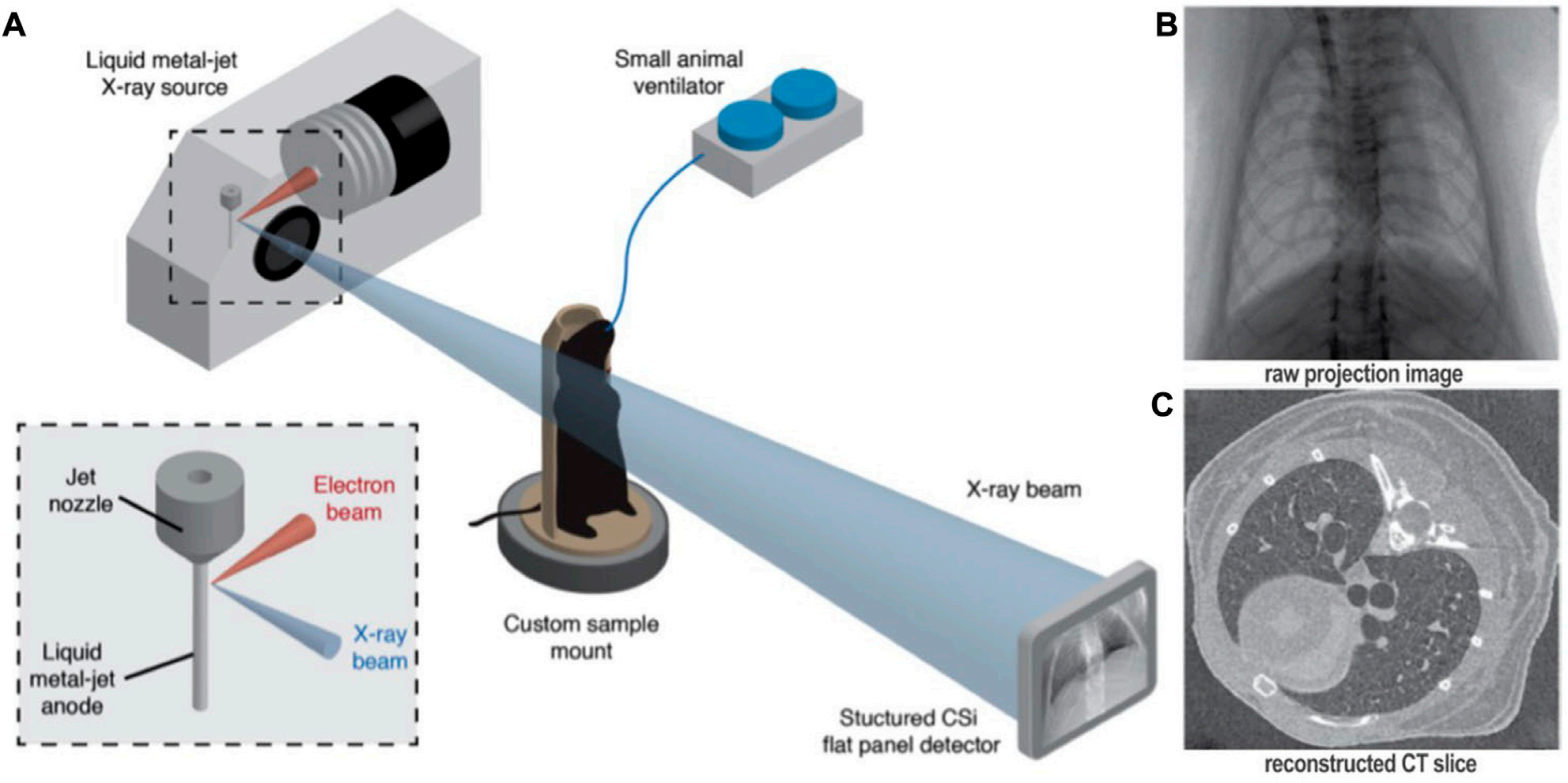
Four-dimensional x-ray velocimetry (4DxV) technique for regional lung function assessments [reprinted from Murrie et al. (2020)]: **(A)** Experimental image acquisition setup using a liquid-metal-jet microfocus X-ray source, introduced by Murrie et al. (2020) for *in vivo* regional lung function measurements; **(B)** Raw 2D projections acquired over 360° using the proposed image acquisition setup; **(C)** Reconstructed CT images from the binned projections to produce 4D CT dataset.

**FIGURE 9:**
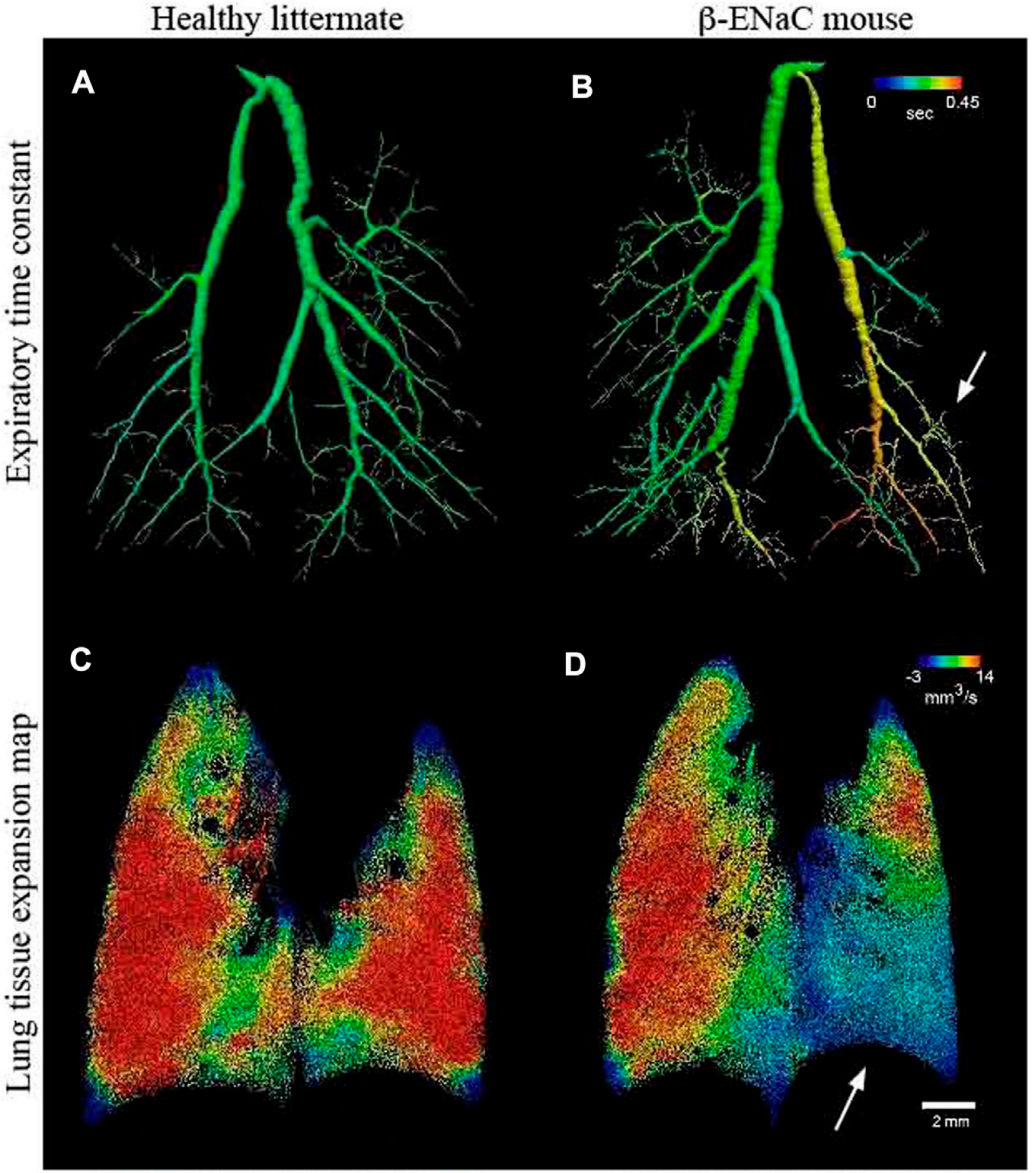
Lung function assessments using 4DxV technique [reprinted from Murrie et al. (Murrie et al., 2020)] **(A, B)** Airway 3D models of a healthy and β-ENaC mice, respectively, colored by local expiratory time constant from 4DxV analysis showing an increased expiratory time constant (arrow) for the β-ENaC mouse compared to the healthy littermate; **(C, D)** Comparison of the lung tissue expantion maps of the healthy and β-ENaC mice, respectively, revealing a reduction in regional tissue expansion (arrow) for the β-ENaC mouse compared to the healthy littermate.

Four-dimensional x-ray velocimetry (4DxV) is a PCXI-based technique that can capture the expansion/contraction of lung tissue throughout a breathing process and also measure the airflow inside the airways. Therefore, any regional structural change of lung parenchyma and alteration of airflow inside the airways due to obstructive/restrictive lung diseases can be detected using this technique (Dubsky et al., 2010; Dubsky et al., 2012; Fouras et al., 2012). Since most of the 4DxV techniques were developed and validated in synchrotron radiation facilities, a more compact and accessible experimental setup was required to make 4DxV mapping more commonly-used in lung research laboratories (Tuohimaa et al., 2007; Bravin et al., 2012; Krenkel et al., 2016). In 2020, Murrie et al. introduced a dynamic *in vivo* 4DxV imaging system using a liquid-metal-jet microfocus X-ray source for regional lung function measurements in β-ENaC mice, a mouse model of cystic fibrosis (CF) (Figure 8) (Murrie et al., 2020). Mice were anesthetized, intubated, and ventilated during scanning. The results of 4DxV analysis, the expiratory time constant, showed a dramatic decrease in regional lung expansion of the left lung for β-ENaC mice in comparison to healthy controls, which correlated directly to the reduction of aeration due to the patchy CF-like airway obstructions in this region. This reduction of aeration directly indicates the regional reduction of lung function for the β-ENaC mouse, which can be considered as a biomarker for early detection of an obstructive airway disease (Figure 9). The proposed 4DxV imaging system is capable of regional imaging of lungs and airways with 60 µm resolution d 30 frames per second for pulmonary functional imaging and obtaining 3D ventilation maps. However, ionizing radiation measurements showed 1.47–1.74 *Gy* radiation dose delivered to each animal during image acquisition, which was below the lethal radiation dose to damage lung tissue (7.5 *Gy* for BALB/C mice and 8.3 *Gy* for C57BL/6 mice (Okunieff et al., 1996)), but still high enough to be considered as the endpoint for the animal.

##### 2.2.2.6 Optical respiratory dynamics tracking

Search for finding a simpler way than WBP and XLF to monitor respiratory dynamics, resulted in introduction of an optical technique by Svetlove et al. called optical respiratory dynamics tracking (ORDT) (Svetlove et al., 2022). This technique is especially useful for tracking diaphragm function of mice with neuromuscular diseases such as Duchenne muscular dystrophy (DMD). ORDT is a simple method for monitoring respiratory dynamics of anesthetized mice using camera tracing of chest surface markers. For developing this optical technique, the authors utilized a camera with the ability to produce images with 600 × 400 pixels resolution for 10 s at 100 frames/sec*.* They placed four paper markers with black cross-hair pattern on the thoracic-abdominal region of the mice with double-sided tape and tracked the movements of these markers in the acquired video with Linear Assignment Problem algorithms. The expiration constant was computed in XLF software. To assess the performance of ORDT technique, the authors used mdx mouse model to investigate the irregularities in the breathing pattern of the mice due to respiratory muscle weakness, which is one of the common characteristics of DMD. In comparison with the data acquired by XLF, the results obtained by ORDT showed significantly steeper expiration for mdx mice compared to the controls by calculating the expiration constant (k), which most probably shows the higher sensitivity of ORDT compared to XLF in capturing the change of respiratory dynamics in dmx mice. Furthermore, unlike XLF technique, ORDT was also able to show the differences between fast and slow expiratory phases in mdx mice, while healthy controls had almost the same fast and slow phases. Compared to the alternative methods for longitudinal assessment of diaphragm function in mice, e. i. WBP and XLF, ORTD is easier to perform, completely non-invasive (no ionizing radiation), cheaper, and can be performed by commonly available tools and equipment (Svetlove et al., 2022). Furthermore, since this optical technique directly assess the dynamics of the body surface, it has a greater potential in detecting abnormal breathing patterns.

##### 2.2.2.7 Pulmonary functional magnetic resonance imaging

In the past decades, alongside CT and nuclear medicine, magnetic resonance imaging (MRI) has been employed to evaluate chronic lung diseases in terms of gas exchange (Ohno et al., 2022). Pulmonary functional imaging with MRI includes measurements of ventilation, perfusion, as well as respiratory motion and mechanics (Wielpütz and Kauczor, 2012), which dates back to 1990s when hyperpolarized (HP) noble gas MR imaging and oxygen-enhanced MRI were introduced for the first time (Albert et al., 1994; Edelman et al., 1996; Kauczor et al., 1998).

Due to the abundance of hydrogen atoms in soft tissues (water and fat protons H1 ) which contain polar nuclei, MRI of lung tissues results in high-quality images. However, in an inflated lung that approximately 80% filled with air, MRI is challenging due to low proton density and abundant air-tissue interfaces, which reduce signal-to-noise ratio and increase magnetic susceptibility effects, resulting in very low inherent signal that is available for lung imaging (Velde et al., 2014; Dubsky and Fouras, 2015; Kumar et al., 2016). Inhalation of HP noble gases by improving the MRI signal overcomes this issue (Dubsky and Fouras, 2015). Contrast agents such as helium-3 ( He3 ) and xenon-129 ( Xe129 ) are the most commonly-used non-radioactive noble gases for HP MRI (Ohno et al., 2022), which have been studied extensively for evaluation of diseases burden and efficacy assessment of therapeutics (Ruppert et al., 2000; Altes et al., 2001; Fain et al., 2006; Fain et al., 2007; Kirby et al., 2012; Chang, 2013). Physical methods of polarization such as spin-exchange optical pumping (SEOP) and metastability exchange (ME) can increase the polarization up to 4-5 order of magnitude above the thermal equilibrium which compensates for the low density of the inhaled noble gas inside the lung (Fain et al., 2010; Adamson et al., 2017). SEOP can be used for polarization of both He3 and Xe129 ; however, ME can be employed only for He3 applications (Adamson et al., 2017).

The biggest advantage of HP He3 MRI is the visualization of areas in the lung which are actively involved in ventilation such as terminal respiratory bronchioles and adjacent alveoli (Santyr et al., 2009; Fain et al., 2010). He3 , with low solubility and a high diffusion coefficient, is the most commonly-used HP noble gas for pulmonary functional imaging due to its large gyromagnetic ratio which offers the strongest signal (Santyr et al., 2009). Due to this unique ability, HP He3 MRI has been used to study many animal models of lung diseases such as asthma, pulmonary fibrosis and emphysema (Holmes et al., 2005; Mata et al., 2007; Thomas et al., 2009; Stephen et al., 2010). For early detection of bleomycin-induced pulmonary fibrosis progression in Sprague-Dawley rats, Stephen et al. employed HP He3 MR imaging and pulmonary function testing by a plethysmography chamber for validation purposes (Stephen et al., 2010). To evaluate lung function and structure of the animals, they used apparent diffusion coefficient (ADC) and fractional ventilation as the most commonly-used metrics. While PFTs showed no significant differences between the treatment groups, fractional ventilation and the ADC value in small airways and alveoli declined for the fibrotic rats, suggesting that the metrics of He3 MRI are more sensitive measures to monitor the progression of pulmonary fibrosis in animal models compared to the parameters of lung function tests. Despite showing the feasibility of measuring the ventilation changes in fibrotic rats, this method had limited success most probably due to the fact that ventilation is an indirect measure of the severity of pulmonary fibrosis (Adamson et al., 2017). In the case of emphysema, it has been shown that He3 ADC is sensitive to alveolar damage due to emphysema in both humans (Salerno et al., 2002; Swift et al., 2005) and animal models (Woods et al., 2004; Jacob et al., 2008; Rodríguez et al., 2009). Also, Xu et al. hypothesized and proved that the transverse diffusion coefficient ( $D_{T}$ ) of He3 apparent diffusion, measured at sub-milli-second diffusion times, is a more sensitive metric for detecting the alveolar damage in the elastase-instillation model of emphysema in rats compared to the longitudinal diffusion coefficient ( $D_{L}$ ) (Xu et al., 2012). Nevertheless, despite many advantages, due to the extremely limited quantity of He3 available worldwide, the cost of using this contrast agent for pulmonary functional imaging is very high which currently limited its application (Fain et al., 2010).

On the other hand, Xe129 as a by-product of the liquid air industry can be found in abundance which consequently leads to a relatively cheaper price compared to He3 (Santyr et al., 2009; Fain et al., 2010). In addition, compared to He3 , Xe129 has higher solubility in tissues and blood which results in a higher ability for quantitative modeling of gas exchange (Kaushik et al., 2013; Qing et al., 2014a; Qing et al., 2014b). To address this issue, due to a lower gyromagnetic ratio and more challenging polarization protocols compared to He3 , the application of HP Xe129 MRI lagged behind (Fain et al., 2010). However, recent protocols for HP Xe129 MRI employs a mixture of Xe with Xe129 isotope enriched up to 85%, which improves the MR signal and solves the problem of low gyromagnetic ratio (Kirby and Parraga, 2013; Mugler and Altes, 2013). In recent years, HP Xe129 MR imaging has been employed in many lung disease studies with different animal models such as radiation-induced lung injury (RILI), allergic inflammation models of asthma, bleomycin models of pulmonary fibrosis, etc. (Cleveland et al., 2014; Doganay et al., 2016; Li et al., 2016; Lilburn et al., 2016; Sharma et al., 2017).

Despite the potential of pulmonary functional MRI (PfMRI) using inhaled hyperpolarized gases ( He3 ; Xe129 ), the development and application of PfMRI have been hampered due to technological challenges such as limited access to MRI and the requirement for multinuclear capabilities which consist of a dedicated Xe129 coil and the polarizer (Kooner et al., 2022). By addressing these technological challenges, further validation, and standardization of imaging protocols and analysis, PfMRI can play an important role in clinical care of many chronic lung diseases such as asthma and interstitial lung diseases to detect symptoms, guide therapy interventions and investigate treatment response (Kooner et al., 2022).

## 3 Toward clinical applications: Translational problems and future perspective of lung function tests

Both invasive and non-invasive conventional methods for lung function measurements, such as plethysmography techniques, have their own advantages and disadvantages (Sections 2.1 and Section 2.2.1). Furthermore, imaging-based techniques for lung function measurements offer non-invasive methods to acquire relevant readouts related to ventilation, perfusion, gas exchange, and lung mechanics (Section 2.2.2). However, almost all of these methods for lung function measurements evaluate the whole lung as a single unit and only offer global readouts. These global lung functional parameters are not sensitive enough to detect the onset and progression of many lung diseases, simply because these pulmonary abnormalities start regionally and their functional effects are masked by lung compensatory mechanisms (Hsia, 2004; Vande Velde et al., 2016; Hsia, 2017; Dekoster et al., 2020). The destructive effects of these lung diseases reflect on global lung functional readouts only after depletion of significant portions of the lung parenchyma. In addition, many of these traditional methods lack the necessary spatial and temporal resolution for the evaluation of lung physiology (Gefter et al., 2021; Ohno et al., 2021). Pulmonary functional imaging is capable of regional quantification of lung physiology as well as pulmonary mechanics with reference to spatial and temporal information derived from time-resolved anatomical imaging (Ohno et al., 2022). Longitudinal evaluation of pulmonary abnormalities on a regional basis in a non-invasive manner is the biggest advantage of pulmonary functional imaging over plethysmography techniques as well as most of the imaging-based techniques for lung function measurements. Pulmonary functional imaging with the capability of capturing regional subtle changes in ventilation even overcomes classical gold-standard techniques, such as FOT, for functional and mechanical assessments of lung. Nowadays, X-ray-CT and MRI by providing high spatial and temporal resolution are the primary imaging modalities for pulmonary functional imaging.

In the past few decades, as pulmonary functional imaging, many non-invasive, novel µCT- and MR-based techniques have been introduced for ventilation and perfusion imaging as well as biomechanics evaluation. On one hand, CT-based methods such as XLF techniques (Section 2.2.2.4), non-invasively offer pulmonary functional readouts related to lung volumes and elasticity in longitudinal *in vivo* animal studies [see (Dullin et al., 2016; Markus et al., 2017; Khan et al., 2021; Dullin et al., 2022)]. In addition, Xe-enhanced ventilation µCT can provide ventilation maps for regional ventilation assessments during the progression of pulmonary diseases with higher spatial resolution than any other imaging modality (Ohno et al., 2021). With further optimization for radiation dose reduction, validation, and standardization of imaging protocols, it is expected that Xe-enhanced ventilation µCT play a more important role in pulmonary functional imaging in near future. Furthermore, PCXI, (Section 2.2.2.5), as a sensitive, high-resolution imaging technique with the capability of providing regional information about airflow and ventilation, as well as structural changes of lung parenchyma showed a promising potential for pulmonary functional imaging (Murrie et al., 2020). With more developments in technology and the availability of *in vivo* 4DxV imaging systems, in near future, PCXI technique can be an inevitable part of every *in vivo* longitudinal animal study for assessment of lung disease models. On the other hand, MR-based techniques such as hyperpolarized noble gas MR imaging using He3 and Xe129 have been widely used for ventilation imaging to evaluate the severity of many chronic pulmonary diseases such as cystic fibrosis, asthma, COPD, etc. [see (Altes et al., 2001; Fain et al., 2006; Thomas et al., 2009; Stephen et al., 2010; Kirby et al., 2012; Cleveland et al., 2014; Doganay et al., 2016; Lilburn et al., 2016)]. Furthermore, oxygen-enhanced MR imaging offers not only valuable information related to regional oxygen enhancement based on oxygen diffusion, but also demonstrates oxygen uptake based on respiration itself. Still, some technical challenges and drawbacks such as dependence on the polarizer and multinuclear technology remain to be overcome to reach the point that these MR-based techniques become fully functional in preclinical and clinical areas (Kooner et al., 2022; Ohno et al., 2022). Considering the future perspective of pulmonary functional imaging using MR, Fluorine-19 ( F19 ) MRI looks like a more promising approach as an economical alternative for hyperpolarized noble gas MRI (17). This technique uses inert fluorinated gases which are non-toxic, inexpensive, and can be found in abundance. In addition, in contrast to hyperpolarized noble gas MRI, F19 MRI can be performed by any MRI scanner with broadband multinuclear imaging capabilities (Couch et al., 2014; Ohno et al., 2022). As the physiological concentrations of detectable mobile fluorine are negligible, it can be a challenge to reach a sufficiently high density of F19 nuclei to label the lung tissue that is required to produce high-quality images (Ruiz‐Cabello et al., 2011; Chapelin et al., 2018). If this technical challenge can be overcome, F19 MRI has great potential to become a feasible and reliable technique for pulmonary functional imaging both in preclinical and clinical areas, but still further validation and optimizations are required.

Since almost all of the pulmonary abnormalities are heterogeneously distributed in the lungs, e.g. interstitial lung diseases, pulmonary functional imaging with the ability to quantify lung functional parameters regionally seems to hold the key for early diagnosis of many lung disorders in the future. Despite the great potential of pulmonary functional imaging for regional lung function assessments, still global conventional spirometric measurements are used in clinics to measure lung volumes, which are not sensitive enough to detect restrictive lung diseases at earliest stages. Several methods of pulmonary functional imaging including Xe129 and F19 MR imaging and Xe-enhanced lung ventilation CT imaging have already shown promising results in the early detection of interstitial lung diseases in humans (Couch et al., 2014; Kong et al., 2014; Ohno et al., 2022). Yet, due to the lack of optimization and standardization of imaging protocols as well as validations in truly clinical trials, these pulmonary functional imaging methods are not translated into the clinics as quantitative tools for monitoring the onset and progression of interstitial lung diseases. Both in clinical and preclinical areas, state-of-the-art lung physiology assessments are gradually, but steadily, shifting toward pulmonary functional imaging using X-ray CT and MRI due to the high temporal and spatial resolution. To accelerate this clinical translation process, interdisiplinary research groups including researchers with different expertise such as pulmonary medicine, imaging, physiology, etc. should be formed to clinically validate the techniques and increase the clinical adoptation of these pulmonary functional methods.

## References

[B1] Adamson E. B. Ludwig K. D. Mummy D. G. Fain S. B. (2017). Magnetic resonance imaging with hyperpolarized agents: Methods and applications. Phys. Med. Biol. 62 (13), R81–R123. 10.1088/1361-6560/aa6be8 28384123 PMC5730331

[B2] Adler A. Cieslewicz G. Irvin C. G. (2004). Unrestrained plethysmography is an unreliable measure of airway responsiveness in BALB/c and C57BL/6 mice. J. Appl. physiology 97 (1), 286–292. 10.1152/japplphysiol.00821.2003 15033965

[B3] Albert M. S. Cates G. D. Driehuys B. Happer W. Saam B. Springer C. S. (1994). Biological magnetic resonance imaging using laser-polarized 129Xe. Nature 370 (6486), 199–201. 10.1038/370199a0 8028666

[B4] Altes T. A. Powers P. L. Knight‐Scott J. Rakes G. Platts‐Mills T. A. E. de Lange E. E. (2001). Hyperpolarized 3He MR lung ventilation imaging in asthmatics: Preliminary findings. J. Magnetic Reson. Imaging An Official J. Int. Soc. Magnetic Reson. Med. 13 (3), 378–384. 10.1002/jmri.1054 11241810

[B5] Amaral J. L. M. Lopes A. J. Jansen J. M. Faria A. C. D. Melo P. L. (2013). An improved method of early diagnosis of smoking-induced respiratory changes using machine learning algorithms. Comput. methods programs Biomed. 112 (3), 441–454. 10.1016/j.cmpb.2013.08.004 24001924

[B6] Amdur M. O. Mead J. (1958). Mechanics of respiration in unanesthetized Guinea pigs. Am. J. Physiology-Legacy Content 192 (2), 364–368. 10.1152/ajplegacy.1958.192.2.364 13508884

[B7] Baelder R. Fuchs B. Bautsch W. Zwirner J. Köhl J. Hoymann H. G. (2005). Pharmacological targeting of anaphylatoxin receptors during the effector phase of allergic asthma suppresses airway hyperresponsiveness and airway inflammation. J. Immunol. 174 (2), 783–789. 10.4049/jimmunol.174.2.783 15634899

[B8] Bates J. Irvin C. Brusasco V. Drazen J. Fredberg J. Loring S. (2004). The use and misuse of Penh in animal models of lung disease. Am. J. Respir. Cell Mol. Biol. 31 (3), 373–374. 10.1165/ajrcmb.31.3.1 15317683

[B9] Bates J. H. T. (2017). Corp: Measurement of lung function in small animals. J. Appl. Physiology 123 (5), 1039–1046. 10.1152/japplphysiol.00243.2017 28798197

[B10] Bates J. H. T. Irvin C. G. (2003). Measuring lung function in mice: The phenotyping uncertainty principle. J. Appl. physiology 94 (4), 1297–1306. 10.1152/japplphysiol.00706.2002 12626466

[B11] Bates J. H. T. Thompson-Figueroa J. Lundblad L. K. A. Irvin C. G. (2008). Unrestrained video-assisted plethysmography: A noninvasive method for assessment of lung mechanical function in small animals. J. Appl. physiology 104 (1), 253–261. 10.1152/japplphysiol.00737.2007 17962577

[B12] Bayat S. Cercos J. Fardin L. Perchiazzi G. Bravin A. (2022). Pulmonary vascular biomechanics imaged with synchrotron phase contrast microtomography in live rats. Switzerland: Eur Respiratory Soc. 10.1038/s41598-022-09052-9 PMC8942151 35322152

[B13] Bayat S. Fardin L. Cercos-Pita J. L. Perchiazzi G. Bravin A. (2022). Imaging regional lung structure and function in small animals using synchrotron radiation phase-contrast and K-edge subtraction computed tomography. Front. Physiology 13, 385. 10.3389/fphys.2022.825433 PMC8957951 35350681

[B14] Bayat S. Le Duc G. Porra L. Berruyer G. Nemoz C. Monfraix S. (2001). Quantitative functional lung imaging with synchrotron radiation using inhaled xenon as contrast agent. Phys. Med. Biol. 46 (12), 3287–3299. 10.1088/0031-9155/46/12/315 11768506

[B15] Bayat S. Porra L. Suortti P. Thomlinson W. (2020). Functional lung imaging with synchrotron radiation: Methods and preclinical applications. Phys. Medica 79, 22–35. 10.1016/j.ejmp.2020.10.001 33070047

[B16] Bayat S. Strengell S. Porra L. Janosi T. Z. Petak F. Suhonen H. (2009). Methacholine and ovalbumin challenges assessed by forced oscillations and synchrotron lung imaging. Am. J. Respir. Crit. care Med. 180 (4), 296–303. 10.1164/rccm.200808-1211OC 19483115

[B17] Berger K. I. (2018). Small airway disease syndromes. Piercing the quiet zone. Ann. Am. Thorac. Soc. 15, S26–S29. 10.1513/AnnalsATS.201710-767KV 29461890 PMC5822397

[B18] Berry G. DeKruyff R. H. Umetsu D. T. Hansen G. (1999). Allergen-specific Th1 cells fail to counterbalance Th2 cell-induced airway hyperreactivity but cause severe airway inflammation. J. Clin. Invest. 103, 175–183. 10.1172/JCI5155 9916129 PMC407883

[B19] Bhattarai P. Myers S. Chia C. Weber H. C. Young S. Williams A. D. (2020). Clinical application of forced oscillation technique (FOT) in early detection of airway changes in smokers. J. Clin. Med. 9 (9), 2778. 10.3390/jcm9092778 32867314 PMC7565456

[B20] Bonnardel E. Prevel R. Campagnac M. Dubreuil M. Marthan R. Berger P. (2019). Determination of reliable lung function parameters in intubated mice. Respir. Res. 20 (1), 211–214. 10.1186/s12931-019-1177-9 31521163 PMC6744631

[B21] Brashier B. Salvi S. (2015). Measuring lung function using sound waves: Role of the forced oscillation technique and impulse oscillometry system. Breathe 11 (1), 57–65. 10.1183/20734735.020514 26306104 PMC4487383

[B22] Bravin A. Coan P. Suortti P. (2012). X-Ray phase-contrast imaging: From pre-clinical applications towards clinics. Phys. Med. Biol. 58 (1), R1–R35. 10.1088/0031-9155/58/1/R1 23220766

[B23] Brennan D. Schubert L. Diot Q. Castillo R. Castillo E. Guerrero T. (2015). Clinical validation of 4-dimensional computed tomography ventilation with pulmonary function test data. Int. J. Radiat. Oncology* Biology* Phys. 92 (2), 423–429. 10.1016/j.ijrobp.2015.01.019 PMC4431937 25817531

[B24] Brown R. H. Walters D. M. Greenberg R. S. Mitzner W. (1999). A method of endotracheal intubation and pulmonary functional assessment for repeated studies in mice. J. Appl. Physiology 87 (6), 2362–2365. 10.1152/jappl.1999.87.6.2362 10601190

[B25] Burgel P-R. Bergeron A. De Blic J. Bonniaud P. Bourdin A. Chanez P. (2013). Small airways diseases, excluding asthma and COPD: An overview. Eur. Respir. Rev. 22 (128), 131–147. 10.1183/09059180.00001313 23728867 PMC9487373

[B26] Buxco D. S. I. (2022). Pulmonary function test. Germany: Springer.

[B27] Cercos-Pita J-L. Fardin L. Leclerc H. Maury B. Perchiazzi G. Bravin A. (2022). Lung tissue biomechanics imaged with synchrotron phase contrast microtomography in live rats. Sci. Rep. 12 (1), 5056–5066. 10.1038/s41598-022-09052-9 35322152 PMC8942151

[B28] Chang Y. V. (2013). Moxe: A model of gas exchange for hyperpolarized 129Xe magnetic resonance of the lung. Magnetic Reson. Med. 69 (3), 884–890. 10.1002/mrm.24304 22565296

[B29] Chapelin F. Capitini C. M. Ahrens E. T. (2018). Fluorine-19 MRI for detection and quantification of immune cell therapy for cancer. J. Immunother. cancer 6, 105–111. 10.1186/s40425-018-0416-9 30305175 PMC6180584

[B30] Chong B. T. Y. Agrawal D. K. Romero F. A. Townley R. G. (1998). Measurement of bronchoconstriction using whole-body plethysmograph: Comparison of freely moving versus restrained Guinea pigs. J. Pharmacol. Toxicol. methods 39 (3), 163–168. 10.1016/s1056-8719(98)00021-5 9741391

[B31] Cleveland Z. I. Virgincar R. S. Qi Y. Robertson S. H. Degan S. Driehuys B. (2014). 3D MRI of impaired hyperpolarized 129Xe uptake in a rat model of pulmonary fibrosis. NMR Biomed. 27 (12), 1502–1514. 10.1002/nbm.3127 24816478 PMC4229493

[B32] Contoli M. Bellini F. Morandi L. Forini G. Bianchi S. Gnesini G. (2016). Assessing small airway impairment in mild-to-moderate smoking asthmatic patients. Eur. Respir. J. 47 (4), 1264–1267. 10.1183/13993003.01708-2015 26869674

[B33] Cosio M. Ghezzo H. Hogg J. C. Corbin R. Loveland M. Dosman J. (1978). The relations between structural changes in small airways and pulmonary-function tests. N. Engl. J. Med. 298 (23), 1277–1281. 10.1056/NEJM197806082982303 651978

[B34] Costa D. L. Tepper J. S. (1988). Approaches to lung function assessment in small mammals. New York: Toxicology of the Lung Raven Press.

[B35] Couch M. J. Ball I. K. Li T. Fox M. S. Ouriadov A. V. Biman B. (2014). Inert fluorinated gas MRI: A new pulmonary imaging modality. NMR Biomed. 27 (12), 1525–1534. 10.1002/nbm.3165 25066661

[B36] Czovek D. (2019). “Pulmonary function tests in infants and children,” in Kendig's disorders of the respiratory tract in children (Netherlands: Elsevier).

[B37] Dane D. M. Yilmaz C. Estrera A. S. Hsia C. C. W. (2013). Separating *in vivo* mechanical stimuli for postpneumonectomy compensation: Physiological assessment. J. Appl. physiology 114 (1), 99–106. 10.1152/japplphysiol.01213.2012 PMC3544515 23104695

[B38] de Andrade Castro J. M. Russo M. (2019). Use and limitations of noninvasive and invasive methods for studying pulmonary function. Drug Discov. Today Dis. Models 29, 3–9. 10.1016/j.ddmod.2019.07.001

[B39] De Langhe E. Vande Velde G. Hostens J. Himmelreich U. Nemery B. Luyten F. P. (2012). Quantification of lung fibrosis and emphysema in mice using automated micro-computed tomography. PLoS One 7, e43123. 10.1371/journal.pone.0043123 22912805 PMC3418271

[B40] De Vleeschauwer S. I. Rinaldi M. De Vooght V. Vanoirbeek J. A. Vanaudenaerde B. M. Verbeken E. K. (2011). Repeated invasive lung function measurements in intubated mice: An approach for longitudinal lung research. Lab. Anim. 45 (2), 81–89. 10.1258/la.2010.010111 21357700

[B41] Dekoster K. Decaesteker T. Berghen N. Van den Broucke S. Jonckheere A-C. Wouters J. (2020). Longitudinal micro-Computed Tomography-derived biomarkers quantify non-resolving lung fibrosis in a silicosis mouse model. Sci. Rep. 10 (1), 16181–16190. 10.1038/s41598-020-73056-6 32999350 PMC7527558

[B42] Dellacà R. L. Santus P. Aliverti A. Stevenson N. Centanni S. Macklem P. T. (2004). Detection of expiratory flow limitation in COPD using the forced oscillation technique. Eur. Respir. J. 23 (2), 232–240. 10.1183/09031936.04.00046804 14979497

[B43] DeLorme M. P. Moss O. R. (2002). Pulmonary function assessment by whole-body plethysmography in restrained versus unrestrained mice. J. Pharmacol. Toxicol. methods 47 (1), 1–10. 10.1016/s1056-8719(02)00191-0 12387933

[B44] Desai J. P. Moustarah F. (2020). Pulmonary compliance. Florida: StatPearls. 30855908

[B45] Devos F. C. Maaske A. Robichaud A. Pollaris L. Seys S. Lopez C. A. (2017). Forced expiration measurements in mouse models of obstructive and restrictive lung diseases. Respir. Res. 18 (1), 123–136. 10.1186/s12931-017-0610-1 28629359 PMC5477381

[B46] Ding K. Bayouth J. E. Buatti J. M. Christensen G. E. Reinhardt J. M. (2010). 4DCT‐based measurement of changes in pulmonary function following a course of radiation therapy. Med. Phys. 37 (3), 1261–1272. 10.1118/1.3312210 20384264 PMC2842288

[B47] Doganay O. Stirrat E. McKenzie C. Schulte R. F. Santyr G. E. (2016). Quantification of regional early stage gas exchange changes using hyperpolarized 129Xe MRI in a rat model of radiation‐induced lung injury. Med. Phys. 43 (5), 2410–2420. 10.1118/1.4946818 27147352

[B48] Donaldson D. D. Dakhama A. Takeda E. W. Rha Y-H. Park J-W. Balhorn A. (2002). The role of IL-13 in established allergic airway disease. J. Immunol. 169, 6482–6489. 10.4049/jimmunol.169.11.6482 12444158

[B49] DuBois A. B. Brody A. W. Lewis D. H. Burgess B. F. Jr (1956). Oscillation mechanics of lungs and chest in man. J. Appl. physiology 8 (6), 587–594. 10.1152/jappl.1956.8.6.587 13331841

[B50] Dubsky S. Fouras A. (2015). Imaging regional lung function: A critical tool for developing inhaled antimicrobial therapies. Adv. drug Deliv. Rev. 85, 100–109. 10.1016/j.addr.2015.03.010 25819486

[B51] Dubsky S. Hooper S. B. Siu K. K. W. Fouras A. (2012). Synchrotron-based dynamic computed tomography of tissue motion for regional lung function measurement. J. R. Soc. Interface 9 (74), 2213–2224. 10.1098/rsif.2012.0116 22491972 PMC3405755

[B52] Dubsky S. Jamison R. A. Irvine S. C. Siu K. K. W. Hourigan K. Fouras A. (2010). Computed tomographic x-ray velocimetry. Appl. Phys. Lett. 96 (2), 023702. 10.1063/1.3285173

[B53] Duguet A. Biyah K. Minshall E. Gomes R. Wang C-G. Taoudi-Benchekroun M. (2000). Bronchial responsiveness among inbred mouse strains: Role of airway smooth-muscle shortening velocity. Am. J. Respir. Crit. care Med. 161 (3), 839–848. 10.1164/ajrccm.161.3.9906054 10712331

[B54] Dullin C. Markus M. A. Larsson E. Tromba G. Hülsmann S. Alves F. (2016). X-Ray based Lung Function measurement-a sensitive technique to quantify lung function in allergic airway inflammation mouse models. Sci. Rep. 6, 36297. 10.1038/srep36297 27805632 PMC5090985

[B55] Dullin C. Svetlove A. Zschüntzsch J. Alves F. (2022). “Improved retrospectively gated *in-vivo* microCT for simultaneous assessment of lung function and anatomy in mice,”. CC BY 4.0. 10.1038/s41598-022-17335-4 PMC9345384 35918439

[B56] Edelman R. R. Hatabu H. Tadamura E. Li W. Prasad P. V. (1996). Noninvasive assessment of regional ventilation in the human lung using oxygen–enhanced magnetic resonance imaging. Nat. Med. 2 (11), 1236–1239. 10.1038/nm1196-1236 8898751

[B57] Ewart L. C. Haley M. Bickerton S. Bright J. Elliott K. McCarthy A. (2010). Pharmacological validation of a telemetric model for the measurement of bronchoconstriction in conscious rats. J. Pharmacol. Toxicol. methods 61 (2), 219–229. 10.1016/j.vascn.2010.02.008 20219687

[B58] Fain S. Schiebler M. L. McCormack D. G. Parraga G. (2010). Imaging of lung function using hyperpolarized helium‐3 magnetic resonance imaging: Review of current and emerging translational methods and applications. J. magnetic Reson. imaging 32 (6), 1398–1408. 10.1002/jmri.22375 PMC3058806 21105144

[B59] Fain S. B. Korosec F. R. Holmes J. H. O'Halloran R. Sorkness R. L. Grist T. M. (2007). Functional lung imaging using hyperpolarized gas MRI. J. Magnetic Reson. Imaging An Official J. Int. Soc. Magnetic Reson. Med. 25 (5), 910–923. 10.1002/jmri.20876 17410561

[B60] Fain S. B. Panth S. R. Evans M. D. Wentland A. L. Holmes J. H. Korosec F. R. (2006). Early emphysematous changes in asymptomatic smokers: Detection with 3He MR imaging. Radiology 239 (3), 875–883. 10.1148/radiol.2393050111 16714465

[B61] Faria A. C. D. da Costa A. A. Lopes A. J. Jansen J. M. de Melo P. L. (2010). Forced oscillation technique in the detection of smoking-induced respiratory alterations: Diagnostic accuracy and comparison with spirometry. Clinics 65 (12), 1295–1304. 10.1590/s1807-59322010001200012 21340218 PMC3020340

[B62] Fay R. R. (1988). Comparative psychoacoustics. Hear. Res. 34 (3), 295–305. 10.1016/0378-5955(88)90009-3 3139607

[B63] Finotto S. De Sanctis G. T. Lehr H. A. Herz U. Buerke M. Schipp M. (2001). Treatment of allergic airway inflammation and hyperresponsiveness by antisense-induced local blockade of GATA-3 expression. J. Exp. Med. 193 (11), 1247–1260. 10.1084/jem.193.11.1247 11390432 PMC2193377

[B64] Flandre T. D. Leroy P. L. Desmecht D. J. M. (2003). Effect of somatic growth, strain, and sex on double-chamber plethysmographic respiratory function values in healthy mice. J. Appl. Physiology 94 (3), 1129–1136. 10.1152/japplphysiol.00561.2002 12571140

[B65] Fouras A. Allison B. J. Kitchen M. J. Dubsky S. Nguyen J. Hourigan K. (2012). Altered lung motion is a sensitive indicator of regional lung disease. Ann. Biomed. Eng. 40 (5), 1160–1169. 10.1007/s10439-011-0493-0 22189492

[B66] Frazer D. G. Reynolds J. S. Jackson M. C. (2011). Determining when enhanced pause (Penh) is sensitive to changes in specific airway resistance. J. Toxicol. Environ. Health, Part A. 74 (5), 287–295. 10.1080/15287394.2010.514235 21240729

[B67] Gattinoni L. D'Andrea L. Pelosi P. Vitale G. Pesenti A. Fumagalli R. (1993). Regional effects and mechanism of positive end-expiratory pressure in early adult respiratory distress syndrome. Jama 269 (16), 2122–2127. 10.1001/jama.1993.03500160092039 8468768

[B68] Gattinoni L. Pelosi P. Crotti S. Valenza F. (1995). Effects of positive end-expiratory pressure on regional distribution of tidal volume and recruitment in adult respiratory distress syndrome. Am. J. Respir. Crit. care Med. 151 (6), 1807–1814. 10.1164/ajrccm.151.6.7767524 7767524

[B69] Gefter W. B. Lee K. S. Schiebler M. L. Parraga G. Seo J. B. Ohno Y. (2021). Pulmonary functional imaging: Part 2—state-of-the-Art clinical applications and opportunities for improved patient care. Radiology 299 (3), 524–538. 10.1148/radiol.2021204033 33847518 PMC8165948

[B70] Glaab T. Daser A. Braun A. Neuhaus-Steinmetz U. Fabel H. Alarie Y. (2001). Tidal midexpiratory flow as a measure of airway hyperresponsiveness in allergic mice. Am. J. Physiology-Lung Cell. Mol. Physiology 280 (3), L565–L573. 10.1152/ajplung.2001.280.3.L565 11159041

[B71] Glaab T. Hoymann H. G. Hohlfeld J. M. Korolewitz R. Hecht M. Alarie Y. (2002). Noninvasive measurement of midexpiratory flow indicates bronchoconstriction in allergic rats. J. Appl. Physiology 93 (4), 1208–1214. 10.1152/japplphysiol.01121.2001 12235016

[B72] Glaab T. Mitzner W. Braun A. Ernst H. Korolewitz R. Hohlfeld J. M. (2004). Repetitive measurements of pulmonary mechanics to inhaled cholinergic challenge in spontaneously breathing mice. J. Appl. physiology 97 (3), 1104–1111. 10.1152/japplphysiol.01182.2003 15121749

[B73] Glaab T. Taube C. Braun A. Mitzner W. (2007). Invasive and noninvasive methods for studying pulmonary function in mice. Respir. Res. 8 (1), 63–10. 10.1186/1465-9921-8-63 17868442 PMC2039738

[B74] Glaab T. Ziegert M. Baelder R. Korolewitz R. Braun A. Hohlfeld J. M. (2005). Invasive versus noninvasive measurement of allergic and cholinergic airway responsiveness in mice. Respir. Res. 6 (1), 139–148. 10.1186/1465-9921-6-139 16309547 PMC1316879

[B75] Goldman M. D. (2001). Clinical application of forced oscillation. Pulm. Pharmacol. Ther. 14 (5), 341–350. 10.1006/pupt.2001.0310 11603948

[B76] Gur D. Drayer B. P. Borovetz H. S. Griffith B. P. Hardesty R. L. Wolfson S. K. (1979). Dynamic computed tomography of the lung: Regional ventilation measurements. J. Comput. assisted Tomogr. 3 (6), 749–753. 10.1097/00004728-197903060-00007 512107

[B77] Gur D. Shabason L. Borovetz H. S. Herbert D. L. Reece G. J. Kennedy W. H. (1981). Regional pulmonary ventilation measurements by xenon enhanced dynamic computed tomography: An update. J. Comput. assisted Tomogr. 5 (5), 678–683. 10.1097/00004728-198110000-00015 7298946

[B78] Hamelmann E. Schwarze J. Takeda K. Oshiba A. Larsen G. L. Irvin C. G. (1997). Noninvasive measurement of airway responsiveness in allergic mice using barometric plethysmography. Am. J. Respir. Crit. care Med. 156 (3), 766–775. 10.1164/ajrccm.156.3.9606031 9309991

[B79] Herbert D. L. Gur D. Shabason L. Good W. F. Rinaldo J. E. Snyder J. V. (1982). Mapping of human local pulmonary ventilation by xenon enhanced computed tomography. J. Comput. assisted Tomogr. 6 (6), 1088–1093. 10.1097/00004728-198212000-00006 6757288

[B80] Hoffman E. A. Ritman E. L. (1985). Effect of body orientation on regional lung expansion in dog and sloth. J. Appl. Physiology 59 (2), 481–491. 10.1152/jappl.1985.59.2.481 4030600

[B81] Hoffman E. A. Tajik J. K. Kugelmass S. D. (1995). Matching pulmonary structure and perfusion via combined dynamic multislice CT and thin-slice high-resolution CT. Comput. Med. Imaging Graph. 19 (1), 101–112. 10.1016/0895-6111(94)00035-2 7736410

[B82] Holmes J. H. Sorkness R. L. Meibom S. K. Sundaram S. K. Perlman S. B. Converse A. K. (2005). Noninvasive mapping of regional response to segmental allergen challenge using magnetic resonance imaging and [F‐18] fluorodeoxyglucose positron emission tomography. Magnetic Reson. Med. An Official J. Int. Soc. Magnetic Reson. Med. 53 (6), 1243–1250. 10.1002/mrm.20504 15906295

[B83] Hoymann H. G. (2007). Invasive and noninvasive lung function measurements in rodents. J. Pharmacol. Toxicol. Methods 55 (1), 16–26. 10.1016/j.vascn.2006.04.006 16793289

[B84] Hoymann H. G. (2012). Lung function measurements in rodents in safety pharmacology studies. Front. Pharmacol. 3, 156. 10.3389/fphar.2012.00156 22973226 PMC3428707

[B85] Hsia C. C. W. (2017). Comparative analysis of the mechanical signals in lung development and compensatory growth. Cell tissue Res. 367 (3), 687–705. 10.1007/s00441-016-2558-8 28084523 PMC5321790

[B86] Hsia C. C. W. (2004). Signals and mechanisms of compensatory lung growth. J. Appl. physiology 97 (5), 1992–1998. 10.1152/japplphysiol.00530.2004 15475557

[B87] Inc S. S. R. E. (2022). FlexiVent for *in vivo* lung function measurements. Canada: SCIREQ.

[B88] Irvin C. G. Bates J. H. T. (2003). Measuring the lung function in the mouse: The challenge of size. Respir. Res. 4 (1), 4–9. 10.1186/rr199 12783622 PMC184039

[B89] Jackson A. C. Watson J. W. (1982). Oscillatory mechanics of the respiratory system in normal rats. Respir. Physiol. 48 (3), 309–322. 10.1016/0034-5687(82)90036-6 7123018

[B90] Jacob R. E. Minard K. R. Laicher G. Timchalk C. (2008). 3D 3He diffusion MRI as a local *in vivo* morphometric tool to evaluate emphysematous rat lungs. J. Appl. Physiology 105 (4), 1291–1300. 10.1152/japplphysiol.90375.2008 PMC2576039 18719237

[B91] Jones A. T. Hansell D. M. Evans T. W. (2003). Pulmonary perfusion quantified by electron-beam computed tomography: Effects of hypoxia and inhaled NO. Eur. Respir. J. 21 (5), 855–861. 10.1183/09031936.03.00085002 12765433

[B92] Kaczka D. W. Ingenito E. P. Suki B. Lutchen K. R. (1997). Partitioning airway and lung tissue resistances in humans: Effects of bronchoconstriction. J. Appl. Physiology 82 (5), 1531–1541. 10.1152/jappl.1997.82.5.1531 9134903

[B93] Kaczka D. W. Lutchen K. R. (2004). Servo-controlled pneumatic pressure oscillator for respiratory impedance measurements and high-frequency ventilation. Ann. Biomed. Eng. 32, 596–608. 10.1023/b:abme.0000019179.87974.7d 15117033

[B94] Kauczor H. U. Surkau R. Roberts T. (1998). MRI using hyperpolarized noble gases. Eur. Radiol. 8 (5), 820–827. 10.1007/s003300050479 9601972

[B95] Kaushik S. S. Freeman M. S. Cleveland Z. I. Davies J. Stiles J. Virgincar R. S. (2013). Probing the regional distribution of pulmonary gas exchange through single-breath gas-and dissolved-phase 129Xe MR imaging. J. Appl. physiology 115 (6), 850–860. 10.1152/japplphysiol.00092.2013 PMC3764620 23845983

[B96] Khan A. Markus A. Rittmann T. Albers J. Alves F. Hülsmann S. (2021). Simple low dose radiography allows precise lung volume assessment in mice. Sci. Rep. 11 (1), 4163–4173. 10.1038/s41598-021-83319-5 33602964 PMC7893164

[B97] King G. G. Bates J. Berger K. I. Calverley P. de Melo P. L. Dellacà R. L. (2020). Technical standards for respiratory oscillometry. Eur. Respir. J. 55 (2), 1900753. 10.1183/13993003.00753-2019 31772002

[B98] Kirby M. Parraga G. (2013). Pulmonary functional imaging using hyperpolarized noble gas MRI: Six years of start-up experience at a single site. Acad. Radiol. 20 (11), 1344–1356. 10.1016/j.acra.2013.02.020 24119346

[B99] Kirby M. Svenningsen S. Owrangi A. Wheatley A. Farag A. Ouriadov A. (2012). Hyperpolarized 3He and 129Xe MR imaging in healthy volunteers and patients with chronic obstructive pulmonary disease. Radiology 265 (2), 600–610. 10.1148/radiol.12120485 22952383

[B100] Kong X. Sheng H. X. Lu G. M. Meinel F. G. Dyer K. T. Schoepf U. J. (2014). Xenon-enhanced dual-energy CT lung ventilation imaging: Techniques and clinical applications. Am. J. Roentgenol. 202 (2), 309–317. 10.2214/AJR.13.11191 24450670

[B101] Kooner H. K. McIntosh M. J. Desaigoudar V. Rayment J. H. Eddy R. L. Driehuys B. (2022). Pulmonary functional MRI: Detecting the structure–function pathologies that drive asthma symptoms and quality of life. Respirology 27 (2), 114–133. 10.1111/resp.14197 35008127 PMC10025897

[B102] Krenkel M. Töpperwien M. Dullin C. Alves F. Salditt T. (2016). Propagation-based phase-contrast tomography for high-resolution lung imaging with laboratory sources. AIP Adv. 6 (3), 035007. 10.1063/1.4943898

[B103] Kumar R. K. Herbert C. Webb D. C. Li L. Foster P. S. (2004). Effects of anticytokine therapy in a mouse model of chronic asthma. Am. J. Respir. Crit. Care Med. 170, 1043–1048. 10.1164/rccm.200405-681OC 15306533

[B104] Kumar S. Liney G. Rai R. Holloway L. Moses D. Vinod S. K. (2016). Magnetic resonance imaging in lung: A review of its potential for radiotherapy. Br. J. radiology 89 (1060), 20150431. 10.1259/bjr.20150431 PMC4846194 26838950

[B105] Lai-Fook S. J. Houtz P. K. Lai Y-L. (2008). End-expiratory and tidal volumes measured in conscious mice using single projection x-ray images. J. Appl. Physiology 104 (2), 521–533. 10.1152/japplphysiol.00729.2007 17872404

[B106] Li H. Zhang Z. Zhao X. Sun X. Ye C. Zhou X. (2016). Quantitative evaluation of radiation‐induced lung injury with hyperpolarized xenon magnetic resonance. Magnetic Reson. Med. 76 (2), 408–416. 10.1002/mrm.25894 26400753

[B107] Li N. Li Q. Bai J. Chen K. Yang H. Wang W. (2020). The multiple organs insult and compensation mechanism in mice exposed to hypobaric hypoxia. Cell Stress Chaperones 25 (5), 779–791. 10.1007/s12192-020-01117-w 32430880 PMC7479670

[B108] Likens S. A. Mauderly J. L. (1982). Effect of elastase or histamine on single-breath N2 washouts in the rat. J. Appl. Physiology 52 (1), 141–146. 10.1152/jappl.1982.52.1.141 6916767

[B109] Lilburn D. M. L. Tatler A. L. Six J. S. Lesbats C. Habgood A. Porte J. (2016). Investigating lung responses with functional hyperpolarized xenon‐129 MRI in an *ex vivo* rat model of asthma. Magnetic Reson. Med. 76 (4), 1224–1235. 10.1002/mrm.26003 PMC5026173 26507239

[B110] Liu X. Salmon P. L. Laperre K. Sasov A. (2017). A comparison study: Image-based vs signal-based retrospective gating on microCT2017. Bellingham: SPIE.

[B111] Lofgren J. L. S. Mazan M. R. Ingenito E. P. Lascola K. Seavey M. Walsh A. (2006). Restrained whole body plethysmography for measure of strain-specific and allergen-induced airway responsiveness in conscious mice. J. Appl. Physiology 101 (5), 1495–1505. 10.1152/japplphysiol.00464.2006 16857859

[B112] Lomask M. (2006). Further exploration of the Penh parameter. Exp. Toxicol. Pathology 57, 13–20. 10.1016/j.etp.2006.02.014 16638630

[B113] Lu Q. Rouby J-J. (2000). Measurement of pressure-volume curves in patients on mechanical ventilation: Methods and significance. Crit. Care 4 (2), 91–100. 10.1186/cc662 11094498 PMC137332

[B114] Lundblad L. K. A. Irvin C. G. Adler A. Bates J. H. T. (2002). A reevaluation of the validity of unrestrained plethysmography in mice. J. Appl. physiology 93 (4), 1198–1207. 10.1152/japplphysiol.00080.2002 12235015

[B115] Lundblad L. K. A. Siddiqui S. Bossé Y. Dandurand R. J. (2021). Applications of oscillometry in clinical research and practice. Can. J. Respir. Crit. Care, Sleep Med. 5 (1), 54–68. 10.1080/24745332.2019.1649607

[B116] MacLeod D. Birch M. (2001). Respiratory input impedance measurement: Forced oscillation methods. Med. Biol. Eng. Comput. 39, 505–516. 10.1007/BF02345140 11712646

[B117] Mailhot-Larouche S. Deschênes L. Lortie K. Gazzola M. Marsolais D. Brunet D. (2018). Assessment of respiratory function in conscious mice by double-chamber plethysmography. JoVE J. Vis. Exp. 137 (137), e57778. 10.3791/57778 PMC6126452 30059019

[B118] Marcucci C. Nyhan D. Simon B. A. (2001). Distribution of pulmonary ventilation using Xe-enhanced computed tomography in prone and supine dogs. J. Appl. physiology 90 (2), 421–430. 10.1152/jappl.2001.90.2.421 11160037

[B119] Markus M. A. Borowik S. Reichardt M. Tromba G. Alves F. Dullin C. (2017). X-ray-based lung function measurement reveals persistent loss of lung tissue elasticity in mice recovered from allergic airway inflammation. Am. J. Physiol. Lung Cell Mol. Physiol. 313 (5), L763–L771. 10.1152/ajplung.00136.2017 28775094

[B120] Marshall R. (1957). The physical properties of the lungs in relation to the subdivisions of lung volume. Clin. Sci. 16 (3), 507–515. 13473164

[B121] Martin T. R. Gerard N. P. Galli S. J. Drazen J. M. (1988). Pulmonary responses to bronchoconstrictor agonists in the mouse. J. Appl. Physiology 64 (6), 2318–2323. 10.1152/jappl.1988.64.6.2318 2457008

[B122] Mata J. F. Altes T. A. Cai J. Ruppert K. Mitzner W. Hagspiel K. D. (2007). Evaluation of emphysema severity and progression in a rabbit model: Comparison of hyperpolarized 3He and 129Xe diffusion MRI with lung morphometry. J. Appl. physiology 102 (3), 1273–1280. 10.1152/japplphysiol.00418.2006 17110518

[B123] Michaelson E. D. Grassman E. D. Peters W. R. (1975). Pulmonary mechanics by spectral analysis of forced random noise. J. Clin. investigation 56 (5), 1210–1230. 10.1172/JCI108198 PMC301985 1184746

[B124] Mitzner W. Tankersley C. (2003). Interpreting Penh in mice. J. Appl. physiology 94 (2), 828–831. 10.1152/japplphysiol.00815.2002 12531918

[B125] Monfraix S. Bayat S. Porra L. Berruyer G. Nemoz C. Thomlinson W. (2004). Quantitative measurement of regional lung gas volume by synchrotron radiation computed tomography. Phys. Med. Biol. 50 (1), 1–11. 10.1088/0031-9155/50/1/001 15715418

[B126] Mori V. Oliveira M. A. Vargas M. H. M. da Cunha A. A. de Souza R. G. Pitrez P. M. (2017). Input respiratory impedance in mice: Comparison between the flow-based and the wavetube method to perform the forced oscillation technique. Physiol. Meas. 38 (6), 992–1005. 10.1088/1361-6579/aa6b75 28378711

[B127] Mugler J. P. Iii Altes T. A. (2013). Hyperpolarized 129Xe MRI of the human lung. J. Magnetic Reson. Imaging 37 (2), 313–331. 10.1002/jmri.23844 PMC3558952 23355432

[B128] Murphy D. J. (2002). Assessment of respiratory function in safety pharmacology. Fundam. Clin. Pharmacol. 16 (3), 183–196. 10.1046/j.1472-8206.2002.00060.x 12165066

[B129] Murrie R. P. Werdiger F. Donnelley M. Lin Y. Carnibella R. P. Samarage C. R. (2020). Real-time *in vivo* imaging of regional lung function in a mouse model of cystic fibrosis on a laboratory X-ray source. Sci. Rep. 10 (1), 447–448. 10.1038/s41598-019-57376-w 31949224 PMC6965186

[B130] Navajas D. Farre R. Rotger M. (1991). “Respiratory impedance,” in Pulmonary function in mechanically ventilated patients. Editors Benito S. Net A. (Berlin, Heidelberg: Springer Berlin Heidelberg).

[B131] Nemery B. Dinsdale D. Verschoyle R. D. (1987). Detecting and evaluating chemical-induced lung damage in experimental animals. Clin. Respir. Physiol. 23 (5), 501–528. 3329920

[B132] Neuhaus-Steinmetz U. Glaab T. Daser A. Braun A. Lommatzsch M. Herz U. (2000). Sequential development of airway hyperresponsiveness and acute airway obstruction in a mouse model of allergic inflammation. Int. archives allergy Immunol. 121 (1), 57–67. 10.1159/000024298 10686510

[B133] Ohno Y. Hanamatsu S. Obama Y. Ueda T. Ikeda H. Hattori H. (2022). Overview of MRI for pulmonary functional imaging. Br. J. Radiology 95 (1132), 20201053. 10.1259/bjr.20201053 PMC9153702 33529053

[B134] Ohno Y. Seo J. B. Parraga G. Lee K. S. Gefter W. B. Fain S. B. (2021). Pulmonary functional imaging: Part 1—state-of-the-art technical and physiologic underpinnings. Radiology 299 (3), 508–523. 10.1148/radiol.2021203711 33825513 PMC8165947

[B135] Okunieff P. Wu T. Huang K. Ding I. (1996). Differential radioprotection of three mouse strains by basic or acidic fibroblast growth factor. Br. J. cancer Suppl. 27, S105–S108. 8763859 PMC2150033

[B136] Olson L. E. Hoffman E. A. (1994). Lung volumes and distribution of regional air content determined by cine X-ray CT of pneumonectomized rabbits. J. Appl. Physiology 76 (4), 1774–1785. 10.1152/jappl.1994.76.4.1774 8045859

[B137] Oostveen E. MacLeod D. Lorino H. Farre R. Hantos Z. Desager K. (2003). The forced oscillation technique in clinical practice: Methodology, recommendations and future developments. Eur. Respir. J. 22 (6), 1026–1041. 10.1183/09031936.03.00089403 14680096

[B138] Özdilek A. (2022). “Lung mechanics-compliance and resistance-extrapulmonary response,” in Teaching pearls in noninvasive mechanical ventilation (Germany: Springer).

[B139] Palecek F. Palecekova M. Aviado D. M. (1967). Emphysema in immature rats: Condition produced by tracheal constriction and papain. Archives Environ. Health An Int. J. 15 (3), 332–342. 10.1080/00039896.1967.10664929 6035083

[B140] Pennock B. E. Cox C. P. Rogers R. M. Cain W. A. Wells J. H. (1979). A noninvasive technique for measurement of changes in specific airway resistance. J. Appl. Physiology 46 (2), 399–406. 10.1152/jappl.1979.46.2.399 422457

[B141] Peslin R. Fredberg J. J. (2011). Oscillation mechanics of the respiratory system. Compr. Physiol., 145–177. 10.1002/cphy.cp030311 23733641

[B142] Poelmans J. Hillen A. Vanherp L. Govaerts K. Maertens J. Dresselaers T. (2016). Longitudinal, *in vivo* assessment of invasive pulmonary aspergillosis in mice by computed tomography and magnetic resonance imaging. Lab. Investig. 96 (6), 692–704. 10.1038/labinvest.2016.45 27019389

[B143] Porra L. Monfraix S. Berruyer G. Le Duc G. Nemoz C. Thomlinson W. (2004). Effect of tidal volume on distribution of ventilation assessed by synchrotron radiation CT in rabbit. J. Appl. Physiology 96 (5), 1899–1908. 10.1152/japplphysiol.00866.2003 14966018

[B144] Powers K. A. Dhamoon A. S. (2019). Physiology, pulmonary ventilation and perfusion. Florida: StatPearls Publishing. 30969729

[B145] Preuss J. M. H. Hall G. L. Sly P. D. (1999). Repeat measurement of respiratory mechanics using the forced oscillation technique in non-paralysed rats. Pulm. Pharmacol. Ther. 12 (3), 173–183. 10.1006/pupt.1999.0198 10419837

[B146] Pride N. B. (1992). Forced oscillation techniques for measuring mechanical properties of the respiratory system. Thorax 47 (4), 317–320. 10.1136/thx.47.4.317 1585299 PMC463717

[B147] Qing K. Mugler J. P. Iii Altes T. A. Jiang Y. Mata J. F. Miller G. W. (2014). Assessment of lung function in asthma and COPD using hyperpolarized 129Xe chemical shift saturation recovery spectroscopy and dissolved‐phase MRI. NMR Biomed. 27 (12), 1490–1501. 10.1002/nbm.3179 25146558 PMC4233004

[B148] Qing K. Ruppert K. Jiang Y. Mata J. F. Miller G. W. Shim Y. M. (2014). Regional mapping of gas uptake by blood and tissue in the human lung using hyperpolarized xenon‐129 MRI. J. magnetic Reson. imaging 39 (2), 346–359. 10.1002/jmri.24181 PMC3758375 23681559

[B149] Ravikumar P. Yilmaz C. Bellotto D. J. Dane D. M. Estrera A. S. Hsia C. C. W. (2013). Separating *in vivo* mechanical stimuli for postpneumonectomy compensation: Imaging and ultrastructural assessment. J. Appl. Physiology 114 (8), 961–970. 10.1152/japplphysiol.01394.2012 PMC3633432 23329819

[B150] Reynolds J. S. Frazer D. G. (2011). Noninvasive pulmonary function screening in spontaneously breathing rodents: An engineering systems perspective. Pharmacol. Ther. 131 (3), 359–368. 10.1016/j.pharmthera.2011.05.003 21635918

[B151] Reynolds J. S. Frazer D. G. (2006). Unrestrained acoustic plethysmograph for measuring tidal volume in mice. Ann. Biomed. Eng. 34 (9), 1494–1499. 10.1007/s10439-006-9159-8 16897419

[B152] Ribeiro C. O. Faria A. C. D. Lopes A. J. de Melo P. L. (2018). Forced oscillation technique for early detection of the effects of smoking and COPD: Contribution of fractional-order modeling. Int. J. Chronic Obstr. Pulm. Dis. 13, 3281–3295. 10.2147/COPD.S173686 PMC6188181 30349233

[B153] Rodríguez I. Pérez‐Sánchez J. M. Peces‐Barba G. Kaulisch T. Stiller D. Ruiz‐Cabello J. (2009). Long‐range diffusion of hyperpolarized 3He in rats. Magnetic Reson. Med. An Official J. Int. Soc. Magnetic Reson. Med. 61 (1), 54–58. 10.1002/mrm.21826 19097202

[B154] Ruiz‐Cabello J. Barnett B. P. Bottomley P. A. Bulte J. W. M. (2011). Fluorine (19F) MRS and MRI in biomedicine. NMR Biomed. 24 (2), 114–129. 10.1002/nbm.1570 20842758 PMC3051284

[B155] Ruppert K. Brookeman J. R. Hagspiel K. D. Mugler J. P. Iii (2000). Probing lung physiology with xenon polarization transfer contrast (XTC). Magnetic Reson. Med. An Official J. Int. Soc. Magnetic Reson. Med. 44 (3), 349–357. 10.1002/1522-2594(200009)44:3<349:aid-mrm2>3.0.co;2-j 10975884

[B156] Salerno M. de Lange E. E. Altes T. A. Truwit J. D. Brookeman J. R. Mugler J. P. (2002). Emphysema: Hyperpolarized helium 3 diffusion MR imaging of the lungs compared with spirometric indexes—initial experience. Radiology 222 (1), 252–260. 10.1148/radiol.2221001834 11756734

[B157] Santyr G. E. Lam W. W. Parra-Robles J. M. Taves T. M. Ouriadov A. V. (2009). Hyperpolarized noble gas magnetic resonance imaging of the animal lung: Approaches and applications. J. Appl. Phys. 105 (10), 102004. 10.1063/1.3112143

[B158] Sauter A. P. Hammel J. Ehn S. Achterhold K. Kopp F. K. Kimm M. A. (2019). Perfusion-ventilation CT via three-material differentiation in dual-layer CT: A feasibility study. Sci. Rep. 9 (1), 5837–5838. 10.1038/s41598-019-42330-7 30967601 PMC6456734

[B159] Schuessler T. F. Bates J. H. T. (1995). A computer-controlled research ventilator for small animals: Design and evaluation. IEEE Trans. Biomed. Eng. 42 (9), 860–866. 10.1109/10.412653 7558060

[B160] Shalaby K. H. Gold L. G. Schuessler T. F. Martin J. G. Robichaud A. (2010). Combined forced oscillation and forced expiration measurements in mice for the assessment of airway hyperresponsiveness. Respir. Res. 11, 82–13. 10.1186/1465-9921-11-82 20565957 PMC2904286

[B161] Sharma S. Narayanasamy G. Przybyla B. Webber J. Boerma M. Clarkson R. (2017). Advanced small animal conformal radiation therapy device. Technol. Cancer Res. Treat. 16 (1), 45–56. 10.1177/1533034615626011 26792490 PMC5616115

[B162] Shinke H. Yamamoto M. Hazeki N. Kotani Y. Kobayashi K. Nishimura Y. (2013). Visualized changes in respiratory resistance and reactance along a time axis in smokers: A cross-sectional study. Respir. Investig. 51 (3), 166–174. 10.1016/j.resinv.2013.02.006 23978643

[B163] Simon B. A. Marcucci C. Fung M. Lele S. R. (1998). Parameter estimation and confidence intervals for Xe-CT ventilation studies: A Monte Carlo approach. J. Appl. physiology 84 (2), 709–716. 10.1152/jappl.1998.84.2.709 9475884

[B164] Simon B. A. (2000). Non-invasive imaging of regional lung function using x-ray computed tomography. J. Clin. Monit. Comput. 16 (5), 433–442. 10.1023/a:1011444826908 12580227

[B165] Simon B. A. (2005). Regional ventilation and lung mechanics using X-Ray CT. Acad. Radiol. 12 (11), 1414–1422. 10.1016/j.acra.2005.07.009 16253853

[B166] Sly P. D. Turner D. J. Collins R. A. Hantos Z. (2005). Penh is not a validated technique for measuring airway function in mice. Am. J. Respir. Crit. care Med. 172 (2), 256. 10.1164/ajrccm.172.2.954 16002576

[B167] Snyder J. V. Pennock B. Herbert D. Rinaldo J. E. Culpepper J. Good W. F. (1984). Local lung ventilation in critically ill patients using nonradioactive xenon-enhanced transmission computed tomography. Crit. care Med. 12 (1), 46–51. 10.1097/00003246-198401000-00013 6360534

[B168] Stephen M. J. Emami K. Woodburn J. M. Chia E. Kadlecek S. Zhu J. (2010). Quantitative assessment of lung ventilation and microstructure in an animal model of idiopathic pulmonary fibrosis using hyperpolarized gas MRI. Acad. Radiol. 17 (11), 1433–1443. 10.1016/j.acra.2010.06.019 20934126 PMC2953546

[B169] Stockley J. A. Cooper B. G. Stockley R. A. Sapey E. (2017). Small airways disease: Time for a revisit? Int. J. chronic Obstr. Pulm. Dis. 12, 2343–2353. 10.2147/COPD.S138540 PMC5557120 28848335

[B170] Su Z-Q. Guan W-J. Li S-Y. Ding M. Chen Y. Jiang M. (2018). Significances of spirometry and impulse oscillometry for detecting small airway disorders assessed with endobronchial optical coherence tomography in COPD. Int. J. chronic Obstr. Pulm. Dis. 13, 3031–3044. 10.2147/COPD.S172639 PMC6171757 30319251

[B171] Svetlove A. Albers J. Hülsmann S. Markus M. A. Zschüntzsch J. Alves F. (2022). Non-invasive optical motion tracking allows monitoring of respiratory dynamics in dystrophin-deficient mice. Cells 11 (5), 918. 10.3390/cells11050918 35269540 PMC8909479

[B172] Swift A. J. Wild J. M. Fichele S. Woodhouse N. Fleming S. Waterhouse J. (2005). Emphysematous changes and normal variation in smokers and COPD patients using diffusion 3He MRI. Eur. J. radiology 54 (3), 352–358. 10.1016/j.ejrad.2004.08.002 15899335

[B173] Tajik J. K. Tran B. Q. Hoffman E. A. (1998). New technique to quantitate regional pulmonary microvascular transit times from dynamic X-ray CT images1998. Bellingham: International Society for Optics and Photonics.

[B174] Takeda K. Hamelmann E. Joetham A. Shultz L. D. Larsen G. L. Irvin C. G. (1997). Development of eosinophilic airway inflammation and airway hyperresponsiveness in mast cell–deficient mice. J. Exp. Med. 186 (3), 449–454. 10.1084/jem.186.3.449 9236197 PMC2198995

[B175] Tarkowski M. Vanoirbeek J. A. J. Vanhooren H. M. Vooght V. D. Mercier C. M. Ceuppens J. (2007). Immunological determinants of ventilatory changes induced in mice by dermal sensitization and respiratory challenge with toluene diisocyanate. Am. J. Physiology-Lung Cell. Mol. Physiology 292 (1), L207–L214. 10.1152/ajplung.00157.2005 16963530

[B176] Taube C. Duez C. Cui Z-H. Takeda K. Rha Y-H. Park J-W. (2002). The role of IL-13 in established allergic airway disease. J. Immunol. 169 (11), 6482–6489. 10.4049/jimmunol.169.11.6482 12444158

[B177] Tepper J. S. Costa D. L. (2015). Methods, measurements, and interpretation of animal lung function in health and disease. Comparative biology of the normal lung. Germany: Elsevier.

[B178] Thomas A. C. Potts E. N. Chen B. T. Slipetz D. M. Foster W. M. Driehuys B. (2009). A robust protocol for regional evaluation of methacholine challenge in mouse models of allergic asthma using hyperpolarized 3He MRI. NMR Biomed. An Int. J. Devoted Dev. Appl. Magnetic Reson. vivo 22 (5), 502–515. 10.1002/nbm.1362 PMC2714734 19204996

[B179] Tielemans B. Dekoster K. Verleden S. E. Sawall S. Leszczyński B. Laperre K. (2020). From mouse to man and back: Closing the correlation gap between imaging and histopathology for lung diseases. Diagnostics 10 (9), 636. 10.3390/diagnostics10090636 32859103 PMC7554749

[B180] Tuohimaa T. Otendal M. Hertz H. M. (2007). Phase-contrast x-ray imaging with a liquid-metal-jet-anode microfocus source. Appl. Phys. Lett. 91 (7), 074104. 10.1063/1.2769760

[B181] Vande Velde G. Poelmans J. De Langhe E. Hillen A. Vanoirbeek J. Himmelreich U. (2016). Longitudinal micro-CT provides biomarkers of lung disease that can be used to assess the effect of therapy in preclinical mouse models, and reveal compensatory changes in lung volume. Dis. Models Mech. 9 (1), 91–98. 10.1242/dmm.020321 PMC4728330 26563390

[B182] Vanoirbeek J. A. J. Rinaldi M. De Vooght V. Haenen S. Bobic S. Gayan-Ramirez G. (2010). Noninvasive and invasive pulmonary function in mouse models of obstructive and restrictive respiratory diseases. Am. J. Respir. Cell Mol. Biol. 42 (1), 96–104. 10.1165/rcmb.2008-0487OC 19346316

[B183] Vanoirbeek J. A. J. Tarkowski M. Ceuppens J. L. Verbeken E. K. Nemery B. Hoet P. H. M. (2004). Respiratory response to toluene diisocyanate depends on prior frequency and concentration of dermal sensitization in mice. Toxicol. Sci. 80 (2), 310–321. 10.1093/toxsci/kfh155 15129019

[B184] Vanoirbeek J. A. J. Tarkowski M. Vanhooren H. M. De Vooght V. Nemery B. Hoet P. H. M. (2006). Validation of a mouse model of chemical-induced asthma using trimellitic anhydride, a respiratory sensitizer, and dinitrochlorobenzene, a dermal sensitizer. J. Allergy Clin. Immunol. 117 (5), 1090–1097. 10.1016/j.jaci.2006.01.027 16675337

[B185] Velde G. V. De Langhe E. Poelmans J. Dresselaers T. Lories R. J. Himmelreich U. (2014). Magnetic resonance imaging for noninvasive assessment of lung fibrosis onset and progression: Cross-validation and comparison of different magnetic resonance imaging protocols with micro–computed tomography and histology in the bleomycin-induced mouse model. Investig. Radiol. 49 (11), 691–698. 10.1097/RLI.0000000000000071 24872004

[B186] Verschakelen J. A. (2010). The role of high-resolution computed tomography in the work-up of interstitial lung disease. Curr. Opin. Pulm. Med. 16 (5), 503–510. 10.1097/MCP.0b013e32833cc997 20644479

[B187] Vijayaraghavan R. Schaper M. Thompson R. Stock M. F. Boylstein L. A. Luo J. E. (1994). Computer assisted recognition and quantitation of the effects of airborne chemicals acting at different areas of the respiratory tract in mice. Archives Toxicol. 68 (8), 490–499. 10.1007/s002040050101 7802589

[B188] Vinogradskiy Y. Y. Castillo R. Castillo E. Chandler A. Martel M. K. Guerrero T. (2012). Use of weekly 4DCT‐based ventilation maps to quantify changes in lung function for patients undergoing radiation therapy. Med. Phys. 39 (1), 289–298. 10.1118/1.3668056 22225299

[B189] Vos R. Ruttens D. Verleden S. E. Vandermeulen E. Bellon H. Vanaudenaerde B. M. (2014). Pregnancy after heart and lung transplantation. Best Pract. Res. Clin. Obstetrics Gynaecol. 28 (8), 1146–1162. 10.1016/j.bpobgyn.2014.07.019 25179291

[B191] Wielpütz M. Kauczor H-U. (2012). MRI of the lung: State of the art. Diagnostic interventional radiology 18 (4), 344–353. 10.4261/1305-3825.DIR.5365-11.0 22434450

[B192] Woods J. C. Yablonskiy D. A. Chino K. Tanoli T. S. K. Cooper J. D. Conradi M. S. (2004). Magnetization tagging decay to measure long‐range 3He diffusion in healthy and emphysematous canine lungs. Magnetic Reson. Med. An Official J. Int. Soc. Magnetic Reson. Med. 51 (5), 1002–1008. 10.1002/mrm.20070 PMC2140251 15122683

[B193] Wu E. Y. Hsia C. C. W. Estrera A. S. Epstein R. H. Ramanathan M. Johnson R. L. Jr (2000). Preventing mediastinal shift after pneumonectomy does not abolish physiological compensation. J. Appl. Physiology 89 (1), 182–191. 10.1152/jappl.2000.89.1.182 10904051

[B194] Xu X. Boudreau M. Ouriadov A. Santyr G. E. (2012). Mapping of 3He apparent diffusion coefficient anisotropy at sub‐millisecond diffusion times in an elastase‐instilled rat model of emphysema. Magnetic Reson. Med. 67 (4), 1146–1153. 10.1002/mrm.23098 22135238

[B195] Young H. M. Eddy R. L. Parraga G. (2019). MRI and CT lung biomarkers: Towards an *in vivo* understanding of lung biomechanics. Clin. Biomech. 66, 107–122. 10.1016/j.clinbiomech.2017.09.016 29037603

